# Emergence and Evolutionary Analysis of the Human DDR Network: Implications in Comparative Genomics and Downstream Analyses

**DOI:** 10.1093/molbev/msu046

**Published:** 2014-01-16

**Authors:** Aida Arcas, Oscar Fernández-Capetillo, Ildefonso Cases, Ana M. Rojas

**Affiliations:** ^1^Computational Cell Biology Group, Institute for Predictive and Personalized Medicine of Cancer, Badalona, Spain; ^2^Genomic Instability Group, CNIO, Madrid, Spain; ^3^Life Sciences Department, Barcelona Supercomputing Center, Barcelona, Spain

**Keywords:** DDR emergence, DDR evolution, DDR posttranslational modifications

## Abstract

The DNA damage response (DDR) is a crucial signaling network that preserves the integrity of the genome. This network is an ensemble of distinct but often overlapping subnetworks, where different components fulfill distinct functions in precise spatial and temporal scenarios. To understand how these elements have been assembled together in humans, we performed comparative genomic analyses in 47 selected species to trace back their emergence using systematic phylogenetic analyses and estimated gene ages. The emergence of the contribution of posttranslational modifications to the complex regulation of DDR was also investigated. This is the first time a systematic analysis has focused on the evolution of DDR subnetworks as a whole. Our results indicate that a DDR core, mostly constructed around metabolic activities, appeared soon after the emergence of eukaryotes, and that additional regulatory capacities appeared later through complex evolutionary process. Potential key posttranslational modifications were also in place then, with interacting pairs preferentially appearing at the same evolutionary time, although modifications often led to the subsequent acquisition of new targets afterwards. We also found extensive gene loss in essential modules of the regulatory network in fungi, plants, and arthropods, important for their validation as model organisms for DDR studies.

## Introduction

Cells are continuously at risk of suffering DNA damage through various exogenous and endogenous insults (Barnes and Lindahl 2004). In humans, the so-called DNA damage response (DDR; Dunham et al. 2012) is activated at DNA breaks, promoting their repair while activating cytostatic or cytotoxic responses that limit the expansion of the damaged cell (Ciccia and Elledge 2010).

Many proteins accumulate at or leave these sites in a dynamic manner, controlled in a precise spatiotemporal manner. DNA damage is initially sensed by specific factors and they are succeeded by the checkpoint factors recruited to the site that cause cell cycle arrest to evaluate the degree of damage. If the damage can be repaired, the necessary components can be brought in once the transcription machinery has left the site, otherwise the cell will undergo apoptosis and die. In conjunction, all these events are largely regulated by posttranslational modifications (reviewed in Polo and Jackson [2011]).

The DDR signaling pathway is a critical guardian of the genome and it is essential to preserve its integrity. Perturbations of this network produce genomic instability, which are inherently related to aging (Fernandez-Capetillo 2010), disease (Ciccia and Elledge 2010), and cancer (reviewed in Lukas [2011]). Thus, certain components of the DDR have been extensively screened for medical purposes. The existence of many druggable protein targets associated with DNA breaks is providing promising opportunities for the development of new therapeutic agents, such as the *ATR* protein (Toledo et al. 2011). The search for druggable targets often focuses on conserved modules within networks given that evolutionary conservation implies some conservation of function and as such, these targets can be easily tested in model organisms. Hence, model species like yeast or flies have been used widely to analyze the function of DDR proteins, for example, the checkpoint kinases (Kazama et al. 2008; Wakabayashi et al. 2008; Stracker et al. 2009) and *ATM* in plants (Kimura and Sakaguchi 2006). Nevertheless, despite the extensive use of model organisms to study DDR, there are still few comprehensive and systematic analyses of the evolutionary conservation of the network as a whole, its regulation or its particular components, with the exception of a few modules like the chromatin modifiers (On et al. 2010).

The information that can be derived from comparative analyses is not only useful to select appropriate targets to address mechanistic or functional aspects of the DDR, and of other networks but also to select the appropriate model organism that best suits specific research purposes. The correct identification of true homologs and/or functionally related proteins is also important to identify precise functional modules, yet it is frequent to find controversial assignations in the literature regarding evolutionary relationships. In particular, through the misassignation of orthology among genes that are evolutionary unrelated but functionally similar (Theissen 2002). Indeed, this is a general and long standing issue in the field of cell biology that seems difficult to overcome (Marabotti and Facchiano 2009).

To fill this gap in our understanding of DDR, to help integrate results from different species, and to guide the selection of the best model organisms for particular studies, we have compiled the first extensive set of consensus human DDR components involved in the sensory, repair, and checkpoint pathways. For the first time, we analyzed their conservation in a wide range of species using methods based on gene age, the classical phylogenetic inference of selected genes, and the emergence and potential conservation of regulatory interactions. Our results revealed a diverse and complex evolutionary history of DDR submodules and a complex pattern through which such pathways have emerged.

## Results

### Classification of DDR Pathways into Four Different Subnetworks

**Fig. 1.**
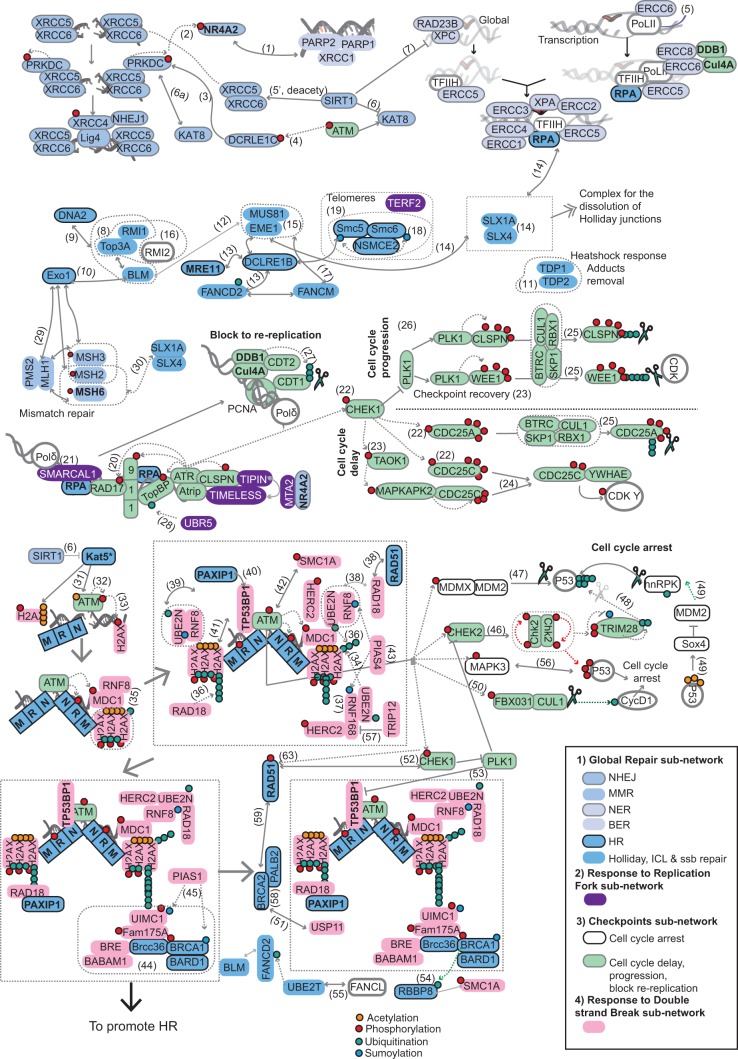
Pathway mapping into the human DDR network. DDR components are classified into four main subnetworks: 1) the GR subnetwork that includes the pathways MMR, BER, NER, HR repair, ssbRep, dissolution of joint DNA molecules (Holliday), ICL, and NHEJ; 2) response at RF subnetwork which includes proteins involved in the sensing and repair of damage at the RF; 3) the CHK subnetwork, and 4) response at DSB subnetwork including sensing the damage at DSB when the NHEJ pathway fails and *ATM*-based repair takes over. This subnetwork overlaps with the HR pathway, and the CHK subnetwork, which includes proteins involved in blocking DNA rereplication, cell cycle delay, cell cycle progression, and related cell cycle arrest as a consequence of response to damage. Interactions of these components are manually extracted from the literature and depicted in this illustration by numbers in brackets that correspond to the PubMed identifiers (supplementary text, Supplementary Material online).

The DDR network encompasses a variety of processes and signals, including repair, sensing, and the activation/resumption of cell cycle checkpoints. To better understand the downstream analyses, we collapsed the human DDR network that contains 13 pathways into four subnetworks (fig. 1), classifying the components accordingly (supplementary table S1, Supplementary Material online). Accordingly, the global response (GR) with 50 components in the subnetwork includes the following pathways: mismatch repair (MMR), base excision repair (BER), nucleotide excision repair (NER), homologous recombination (HR), single strand break repair (ssbRep), dissolution of joint DNA molecules (or Holliday junctions), interstrand-cross link (ICL) repair, and nonhomologous end joining (NHEJ). The response at replication Forks (RF) subnetwork includes nine components involved in the response to damage at the RF. The response to double strand breaks (DSB) subnetwork contains 17 components involved in sensing the damage at DSB when NHEJ fails and *ATM*-based repair takes over. The checkpoints (CHK) subnetwork includes 30 proteins involved in blocking DNA rereplication, cell cycle delay, cell cycle progression, and cell cycle arrest as a consequence of response to damage. Among these subnetworks, there is some degree of overlap as certain DDR components act in different pathways from different subnetworks (e.g., some components recruited to the foci upon response to DSB also promote further HR: supplementary table S1, Supplementary Material online). There are 11 overlapping components (fig. 1), of which seven are common proteins for the DSB and GR (HR pathway) subnetworks; two for the CHK (block to rereplication pathway) and GR (NER pathway) subnetworks; one component belongs to the GR subnetwork (BER and NHEJ pathways) and the RF subnetwork; and another one belongs to the GR (HR pathway) and RF subnetwork (supplementary table S1, Supplementary Material online).

### Global Analyses of the DDR network

#### Phylogenetic Profile of DDR Proteins

**Fig. 2.**
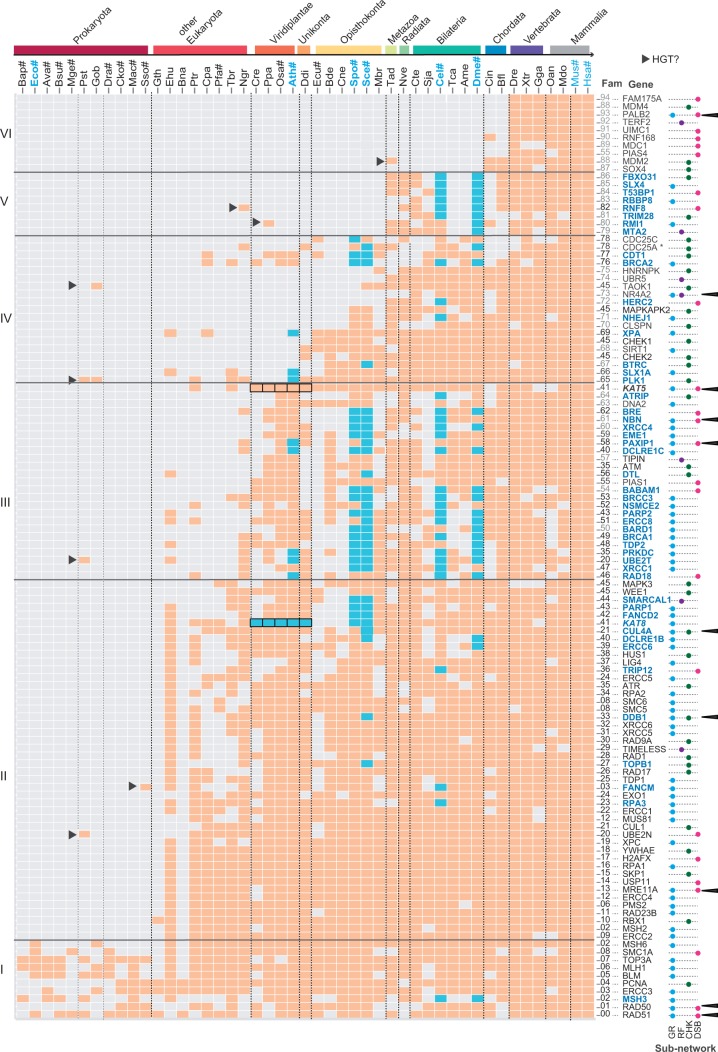
Phylogenetic profile of DDR proteins. (Top) A three-code name corresponds to the species (the full species names are available in supplementary table S2, Supplementary Material online), where blue indicates frequently used model species and # indicates completely sequenced genomes. The color bars over the names indicate the species included in the particular age groups. (Right) The names correspond to human DDR genes retrieved from the literature. Blue names indicate the absence of these genes in model species. The numbers associated with the names are family identifiers and the numbers in gray indicate proteins where phylogenetic analyses have not been carried out. Colored dots indicate assigned DDR subnetworks. In the matrix, orange boxes indicate the presence of homologs, while gray boxes indicate their absence, and the blue boxes indicate absences in completely sequenced model organisms having a homolog in earlier species. The black triangles indicate homologs found in only a few species, where horizontal gene transfers may have occurred. Black arrows at the right of the names indicate when a given gene is involved in more than one pathway. The black stroke in the *KAT8* and *KAT5* genes indicates the misassignation of orthology due to the automated process. The figure was generated with ggplot2 R library (Wickham 2009).

After careful inspection of the available literature, we manually selected 118 human proteins (supplementary table S2, Supplementary Material online), and we used these to search for homologs in 46 species that cover a significant range of the evolutionary tree that would help us pinpoint the evolutionary age of each particular protein (supplementary table S1, Supplementary Material online). The presence or absence of homologs in each of the 47 species was used to construct a phylogenetic profile (fig. 2) for each of them. Six distinguishable blocks were identified, the first of which included ten proteins conserved in all three kingdoms and they were mainly involved in the GR subnetwork pathways (blue dots on names in fig. 2): mismatch—*MLH1*, *MSH3*, and *MSH6*; dissolution of Holliday junctions—*TOP3A*, *BLM*, and *FANCM;* and *PCNA* represents the CHK subnetwork. No components of the RF subnetwork were found. The second block included 46 proteins conserved within all eukaryotes with a few absences in some organisms. These proteins represent components of all the subnetworks (GR, DSB, RF, and CHK), although the majority of these components were still from the GR subnetwork. The same applies to the third block, which included 25 proteins that appeared before the Viridiplantae split not identified in early eukaryotes. The fourth block included 19 proteins that are mostly from the CHK subnetwork. The fifth block included eight proteins from basal metazoans (*Trichoplax adhaerens* and/or *Nematostella vectensis*) and finally the sixth block included ten proteins with a relevant role in the DSB subnetwork that were only found in Chordata. These last two blocks included components from all subnetworks.

There were other proteins that appeared to be incorrectly situated, and that appeared to be shuffled between blocks. For instance, *BRCA2* and *CDT1* (block IV) are present in plants and thus, it would be expected to find them in block III. However, as they are also present in most animals, the automatic clustering grouped them in block IV. In addition, many proteins were found as single instances in a single age group (black triangles, fig. 2), such as *RNF8* in *Naegleria gruberi*, or *UBE2T* in the bacterium *Pirellula staleyi*. In some cases, different components act on more than one pathway (black arrows at the right of the names, fig. 2).

#### The Emergence of the DDR Proteins

**Fig. 3.**
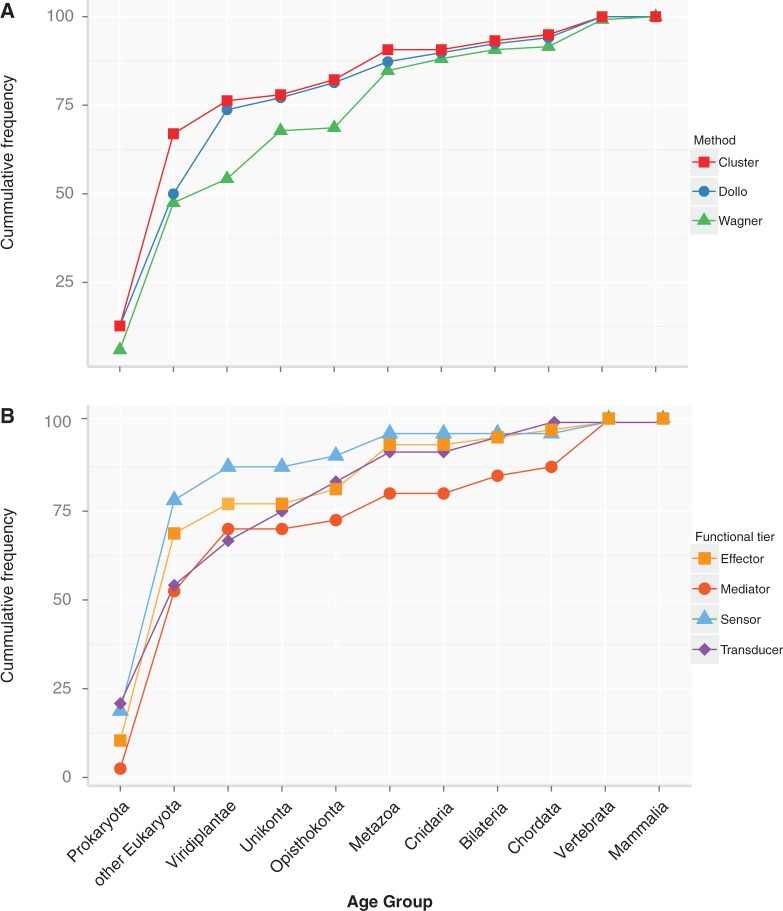
Emergence of DDR proteins. The plots indicate the cumulative frequency of the proteins (*Y* axis) in each age group (*X* axis) and they were generated with the ggplot2 R library (Wickham 2009). (A) Emergence of total components. The red line represents the path according to the hierarchical clustering of the phylogenetic profile, while the blue and green plots are the paths estimated by Dollo and Wagner parsimony, respectively. (B) Emergence of proteins according to functional tiers. The plot illustrates the emergence of different classes according to the different functional classifications (supplementary table S1, Supplementary Material online), as obtained by hierarchical clustering.

We assigned evolutionary ages to the genes of the DDR network and quantified the number of genes that emerged at each evolutionary age. According to the phylogenetic profile ordered by evolutionary timing (fig. 3*A*), around 10% of the human proteins could be traced to the Prokaryota group that included archaea and bacteria. Although the RF component *UBR5* was identified in one bacterial species, the earliest hit occurred in animals, indicative of either a genomic artifact or lateral gene transfer. Further screening in 41 additional prokaryotic genomes (data not shown) confirmed this observation, so we can safely assume that components of the RF pathway were not present in prokaryotes.

By the time the Eukaryota split occurred, there had been a large expansion of genes, whereby most of the DDR components seem to have been acquired (∼70%), most of which belonged to the GR subnetwork (fig. 2, blue dots on names). The remaining subnetworks also emerged at this stage.

From this point on, the incorporation of novel components seemed less remarkable with the exception of the incorporation of many CHK components at Opisthokonta (fig. 2, green dots). This incorporation of genes had been fully established by the time the Vertebrata group appeared, with no subsequent innovations detected.

Two other more sophisticated methods to infer gene age, Wagner and Dollo parsimony (Csuros 2010), agreed well with the simple clustering data (fig. 3*A*), although there were some differences in the relative numbers as was expected given that both methods account differently for gene loss or gain (fig. 3*A*). Gene age enrichment analyses of our human data set was performed using different reference databases, and both these parsimony-based methods (Dollo and Wagner) also showed a significantly enrichment in genes originated with the eukaryotic lineage and a significant underrepresentation in mammalian-specific genes (table 1).

**Table 1.** Age Enrichment for the Human DDR Using Different Algorithms.

DB^a^	A^b^	*N*^c^	O^d^	U^e^	AVGi^f^	AVGb^g^	Mi^h^	Mb^i^	Mann–Whitney *U* Test
Jaccard	W	12	E***	B***	1,372.9	1,138.9	1,628	910	*U* = 9.5e + 05 (*P* = 0.000147)
Multiparanoid	W	12	E***/O**	H**	1,103.7	797.8	910	454.6	*U* = 8.6e + 05 (*P* = 2.4e−07)
Panther7	W	38	E***/O***	—	1,224.7	681.4	910	454.6	*U* = 6.7e + 05 (*P* = 2.22e−16)
OthoMCL	W	12	E***/O***	H**	1,079.1	639.6	910	454.6	*U* = 7.3e + 05 (*P* = 2.19e−13)
Nens	W	12	E***/O*	H**	1,296.5	947.8	1,368	454.6	*U* = 8.6e + 05 (*P* = 1.49e−07)
Jaccard	D	12	E***	B***	1,556.2	1,289.1	1,628	910	*U* = 9e + 05 (*P* = 4.78e−06)
Multiparanoid	D	12	E***	M**	1,360.7	959.5	1,628	454.6	*U* = 7.7e + 05 (*P* = 5.76e−11)
Panther7	D	38	E***	Eu***	1,764.3	1,154.5	1,628	910	*U* = 6.5e + 05 (*P* = 0)
OthoMCL	D	12	E***/O**	M**	1,345.5	817.9	1,628	454.6	*U* = 6.5e + 05 (*P* = 0)
Nens	D	12	E***	M**/H*	1,532.6	1,107.8	1,628	910	*U* = 7.8e + 05 (*P* = 6.12e−11)

^a^Database is HUMAN_PPODv4 clustered with the corresponding method.

^b^Algorithm: W is Wagner and D is Dollo.

^c^*N*: Number of species in species tree.

^d^O is Overrepresented (E: Eukaryota and O: Opisthokonta).

^e^U is Underrepresented (M: Mammals, H: Human, Eu: Euteleostomi, and B: Bilateria).

^f^AVGi: average age input set.

^g^AVGb: average age background set.

^h^Mi: median input data set.

^i^Mb: median background. Fisher’s exact test was used to calculate the significance of the differences for each age group: *P < 0.05; **P < 0.01; ***P < 0.001. For details of the algorithms and databases please check http://lighthouse.ucsf.edu/ProteinHistorian (last accessed January 28, 2014).

#### Emergence of the DDR Proteins According to Functional Criteria

Using a four-tier functional classification (see Materials and Methods), the most populated classes in *Homo sapiens* were the effectors (48 proteins), followed by the mediators (40 proteins), the sensors (32 proteins), and finally, the transducers (24 proteins). This functional classification overlaps to some extent with that reported in different studies, as the same protein may share different functions (supplementary table S1, Supplementary Material online). We plotted the emergence of the different functions along the evolutionary scale and as might be expected, the ancestral core (block I in the profile, fig. 2) contained all four functional classes, with transducers and sensors being the most abundant. From here, each of the classes followed different patterns. Effectors and sensors accumulated early, with 69% and 79% of them having homologs in early eukaryotes, whereas this figure was less than 55% for mediators and transducers. By the time Metazoa emerged, more than 90% of the sensors, transducers, and effectors had appeared, whereas only 80% of the mediators could be detected. Indeed, up to 13% of this latter class of proteins is vertebrate specific (fig. 3*B*).

The distribution of these functional tiers in the different subnetworks is variable (supplementary table S1, Supplementary Material online). In terms of nonoverlapping components (those that act exclusively in one pathway), effectors dominate in the GR and CHK subnetworks, while mediators dominate in the DSB and RF subnetworks. With the exception of the GR subnetwork, sensors are generally poorly represented. Transducers are widely represented in both DSB and CHK subnetworks, while they are depleted in the GR and RF subnetworks. For the ten DDR components that are associated with more than one subnetwork (fig. 2, black arrows), all four functions can also be found.

### Evolutionary Conservation of DDR Subnetworks

**Fig. 4.**
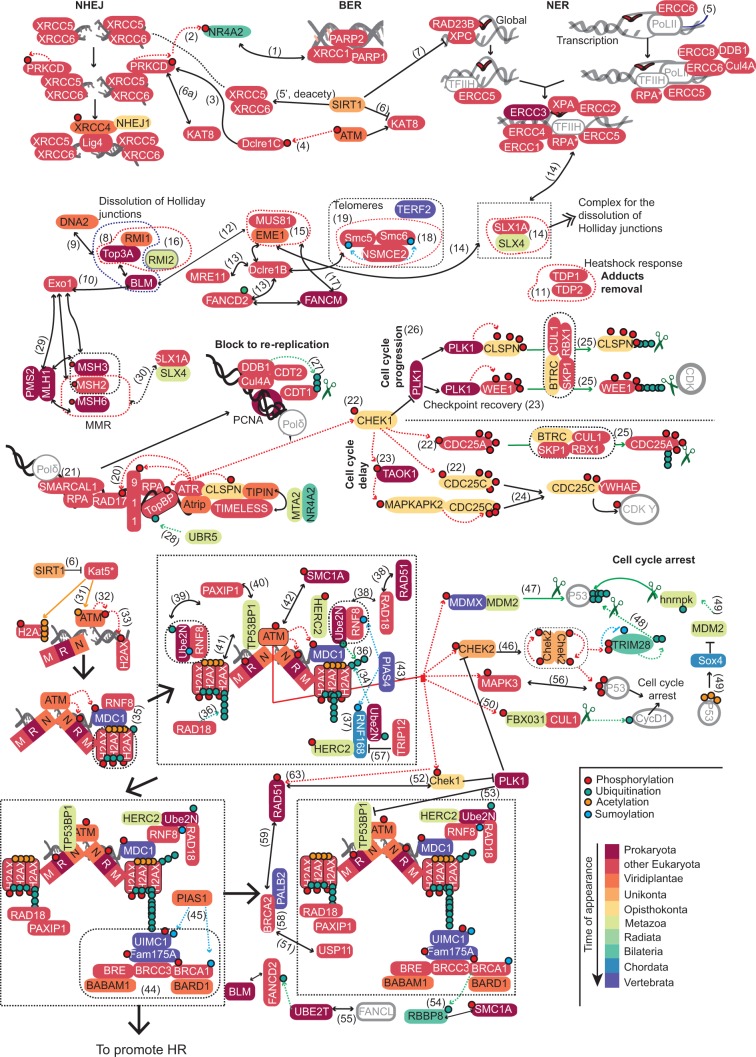
Evolutionary conservation of DDR components in the human DDR network. The colors (from reddish in the older genes to blue in the newer ones) represent the conservation measured as the presence in at least one species of the age group, as in figure 2. Small circles: green, ubiquitination; red, P; yellow, acetylation; and blue, sumoylation. The dotted boxes indicate protein complexes and the numbers in brackets correspond to the PubMed identifiers as in figure 1 (supplementary text, Supplementary Material online).

The evolutionary conservation of the DDR subnetworks (fig. 2) has been mapped (fig. 4) to the human network.

#### The GR Subnetwork and Its Pathways

##### Nonhomologous End Joining.

This is the error-prone repair pathway that is most deleterious because it does not use information from the undamaged DNA template. In this process, the *XRCC5/6* heterodimer binds to DNA ends to protect them from resection, and then, *PRKDC* is recruited and activated by autophosphorylation, bridging the two proximal ends (Lees-Miller and Meek 2003)*.* If end processing is required, the *XRCC5/6*-*PRKDC* complex binds to *DLCRE1C*, activating its endonuclease activity (Kurosawa and Adachi 2010). Finally, the end-ligation step is mediated by the *LIG4-XRCC4* complex, which associates with *NHEJ1* (Hentges et al. 2006). The NHEJ pathway is further regulated by acetylation/deacetylation, whereby *PRKDC* can associate to *KAT8* (acetylase: Sharma et al. 2010), which can in turn be deactivated by the *SIRT1* deacetylase (Peng et al. 2012).

None of these components were detected in prokaryotes, with *XRCC5*/*6*, *PRKDC*, *DLCRE1C,* and *KAT8* were identified after the eukaryote explosion. Sequential incorporation commenced with *XRCC4* before the plants split, the deacetylase *SIRT1* that emerged in Unikonta, and *NHEJ1* that appeared even later, at the Opisthokonta split. The NHEJ pathway was completed before the split of animals and fungi.

##### Base Excision Repair.

Small chemical alterations like alkylation, deamination, abasic or AP sites, single strand breaks, and oxidation of DNA bases can be corrected by excision of the damaged base, the incorporation of the correct nucleotide(s) and strand ligation (Ciccia and Elledge 2010). None of the core components of BER, such as *PARP1*, *PARP2,* and *XRCC1* were identified in prokaryotes, although they had all emerged in early eukaryotes. Regulation of this complex by *NR4A2* appeared later, before bilaterians emerged, yet the pathway was completed in early eukaryotes.

##### Nucleotide Excision Repair.

The NER system recognizes bulky and helix-distorting base pair lesions. Its global genome version scans the entire genome, where the *XPC-RAD23B* complex detects the lesions that open DNA and recruiting transcription factor IIH (*TFIIH*). In the transcription-coupled mode, the damage is sensed by the stalled RNA polymerase II, and by *ERCC8* and *ERCC6* (Fousteri and Mullenders 2008). Both these routes create short stretches of ssDNA that is stabilized by *XPA* and the HR component *RPA* (discussed in the HR section). Subsequently, *ERCC3* and *ERCC2* (the two helicase subunits of *TFIIH*) bind to and extend the ssDNA, before the endonucleases *ERCC4-ERCC1* and *ERCC5* cleave the 5′ and 3′ sides of the lesion, that is then filled in by *PCNA*, *RPA,* and DNA polymerases. The gap is finally sealed by DNA ligases I/III.

The helicase *ERCC3* was the only component detected at the ancestral core. However, by early eukaryotes the complete pathway had already been assembled, with *ERCC1*, *ERCC2*, *ERCC4*, *ERCC5,* and *XPA*, as components of the global pathway, with *RAD23B* and *XPC*, and also with the elements involved in the NER coupled to transcription *ERCC6* and *ERCC8*.

##### Dissolution of Joint DNA Molecules, Single Strand Breaks Repair, and ICL Repair.

The joining of DNA molecules (also known as Holliday junctions) are mobile junctions between several DNA strands, and complexes of *RMI1/TOP3A/BLM* (Raynard et al. 2006) or *RMI1/TOP3A/RMI2* (Singh et al. 2008) must be assembled to dissociate these structures. Of these, *BLM* and *TOP3A* (also *TOP2A*, not included in this study) constitute the ancestral core and they were identified in prokaryotes. The other components appeared later, where *RMI1* (before the *Viridiplantae* split) and *RMI2* (in animals) completed the assembly of the complexes.

Other proteins important for Holliday junctions’ dissolution are the structure-specific endonucleases *EME1/MUS81* and *SLX1A/SLX4* (Fekairi et al. 2009). Moreover, the exonuclease *DCLRE1B* interacts with many proteins (fig. 1), in particular with the *EME1/MUS81 complex*. These complexes were at least partially evident in early eukaryotes (*MUS81* and *SLX1A*), as well as *DCLRE1B*. However, *EME1* and the regulatory unit of *SLX4* appeared later in animals. Thus, our results indicate that this pathway has been assembling during the evolution. This repair pathway has been frequently associated with proteins involved in repairing single-stranded DNA, which are the *TDP1/2* components. All of which were identified in early eukaryotes stage.

ICL is another form of damage (Bae et al. 2008) and the elements involved in ICL repair have been present since prokaryotes, which contain both *UBE2T* and *FANCM*. By contrast, *FANCD2* emerged later at early eukaryotes.

##### Homologous Recombination.

This is the error-free repair system that commences with the MRN-complex (*MRE11A–RAD50–NBN*) recognizing and binding to breaks, holding together and stabilizing the DNA ends (Williams et al. 2007), and serving as a scaffold to promote end resection. The components involved in this process are the *RBBP8* endonuclease, the *BLM* helicase, the *DNA2* helicase/endonuclease and *EXO1.* Subsequently*, RPA* binds to the overhanging ends and this protein is then replaced by *RAD51* (mediated by *BRCA2* and *PALB2*), which promotes the search for the homologous duplex DNA in the undamaged sister chromatid, and facilitates strand invasion into the homologous template.

Given the overlapping with the DSB repair subnetwork, the central MRN complex is presented later. Although *EXO1*, *RPA, RAD51,* and *BRCA2* were all identified in early eukaryotes, *RBBP8* appeared later in bilaterians and *PALB2* much later, before vertebrates emerged.

At the telomeres, HR is performed by a complex including *SMC5/6* and *NSMCE2* (Potts and Yu 2007), and it is also mediated by the exonuclease *DCLRE1B* (discussed earlier; Ye et al. 2010), all proteins that were identified in early eukaryotes. However, this complex may be regulated via *TERF2* (Kim et al. 2009), a recent form of modulation as *TERF2* can only be identified in vertebrates.

##### Mismatch Repair.

This route eliminates mismatches that arise during DNA replication. In eukaryotes, these events are recognized by the MutSα heterodimers (*MSH2/MSH6*), which binds base–base mismatches and small insertion–deletion loops, and the MutSβ heterodimers (*MSH2/MSH3*) that bind larger ones. The heterodimer MutLα (*MLH1/PMS2*) is recruited by the *MSH2* protein to form a ternary complex with one of the MutS complexes and it promotes repair via its endonucleolytic activity, leading to excision repair of the mismatch (Kadyrov et al. 2006). Additional proteins involved in this process may include *EXO1*, *RPA* (an HR component), *PCNA* (a checkpoint component), and DNA polymerases α and β (Li 2008). The oldest members of this pathway are *MSH3*, *MSH6*, and *MLH1*, one of the subunits in each of the complexes (fig. 1), and these were detected in prokaryotes. In early eukaryotes, the *MSH2* subunit emerged that is responsible for recruiting MutL to the mismatches, and once *PMS2* emerged, the MMR pathway was complete and it has remained unchanged since.

#### The Response at RF Subnetwork

In this subnetwork, *ATR* is activated when ssDNA is generated at stalled RF or due to the processing of DSB ends. There, the HR replication protein A (*RPA*) binds to the newly created ssDNA overhangs and it recruits *SMARCAL1*, an ATP-dependent annealing helicase involved in the replication stress response (Bansbach et al. 2009). Only *SMARCAL1* and *RPA* (described earlier, in HR) have been identified at early eukaryotes.

Additional checkpoint components are recruited to these sites (discussed later in the CHK subnetwork), and additional regulation is exerted by the E3-ubiquitin ligase *UBR5*, the transcription factor *MTA2* and *NR4A2*. These latter components were acquired at different steps and while *MTA2* and *UBR5* appeared in Metazoa, NR4A2 emerged later in *Bilateria* to complete the regulatory elements of this assembly. The evolutionary conservation of the remaining components (from the CHK pathway) is explained in corresponding sections.

#### The Checkpoints Subnetwork (CHK) and Its Pathways

These pathways emerged to estimate the degree of damage in replication and the cell cycle, producing a block in rereplication, cell cycle progression/delay, or cell cycle arrest. Checkpoints are important to drive the cell through the cell cycle and this is mediated by the *CDC25* family of dual-specificity phosphatases (especially at the G1/S transition), which are inactivated by *ATM/CHEK2* and *ATR/CHEK1* (Reinhardt and Yaffe 2009). When the DDR network is triggered, *PLK1* is inhibited, thereby blocking cell-cycle progression at the G2/M transition. In the normal G2/M transition, *PLK1* inhibits *CHEK2* and *TP53BP1* (van Vugt et al. 2010).

##### Block of DNA Rereplication.

DDR participates in the inhibition of DNA replication where DNA is damaged, partly by targeting the *CDT1* protein for ubiquitin-mediated destruction (Arias and Walter 2007). Blocking DNA rereplication is mediated by *PCNA* (already present in Archaea), whereas all the remaining components were identified in early eukaryotes, *DTL*, *CDT1, CUL4A,* and *DDB1*; the latter two participating in the NER pathway coupled to transcription.

##### Cell Cycle Delay and Progression.

*ATR* activation depends on *RAD17* loading of the 9-1-1 complex onto DNA (*RAD9-RAD1-HUS1*), where the interaction of *ATR* with *ATRIP* regulates the checkpoint response (Cimprich and Cortez 2008). Further recruitment of *TOPBP1, CLSPN*, *TIMELESS,* and *TIPIN*, promote *ATR* phosphorylation, as well as the activation of *CHEK1*, and other kinases like *TAOK1* and *MAPKAPK2* (Reinhardt and Yaffe 2009), which in turn control certain checkpoints (Allen et al. 2011).

Only *TAOK1* and *PLK1* homologs were identified in the ancestral core, in *Planctomycetes* species (see Discussion), and they are involved in cell cycle delay and progression, respectively. However, in early eukaryotes, the checkpoint network expanded remarkably with the introduction of genes regulating the cell cycle. Thus, *HUS1*, *RAD1*, *RAD9* (the three that form the 9-1-1 complex), *ATR*, *RAD17*, *TOPBP1*, *CDC25A*, *RBX1*, *CUL1*, *SKP1,* and *YWHAE* emerged as components of this subnetwork. *ATRIP* emerged later, before the Viridiplantae split. However, the essential regulators triggering this activation, *CHEK1* and *CLSPN,* appeared even later, before the split of fungi and animals, with the subsequent recruitment of additional members, for instance *BTRC*. At the same time, alternative routes to reinforce cell cycle delay emerged through the appearance of *CDC25C* and *MAPKAPK2*. This pathway was then completely assembled before the split of fungi and animals.

##### Cell Cycle Arrest.

The control of cell cycle arrest is mediated by *ATM*, which appeared later at plants, although the activator of the pathway is *CHEK2*, which appeared a bit later and was identified in amoebas. *ATM-*activated *CHEK2* regulates *P53*, which induces cell-cycle arrest, senescence or apoptosis in response to DNA damage (Ahn et al. 2004). *ATM* also phosphorylates *FBXO31*, which triggers the ubiquitination and subsequent degradation of cyclin *D1* by the proteasome, resulting in G1 arrest after DNA damage (Santra et al. 2009).

A much later connection with cell cycle arrest pathways involving *P53* was established with the emergence of *MDM2*, *FBX031,* and *HNRNPK* in Metazoa, and *TRIM28* in bilaterians. These new components were connected to the oldest elements of the pathway, *MAPK3* and *CUL1*, which were already present in early eukaryotes, or to the newest members, *MDMX* and *SOX4* that emerged in Vertebrata or Chordata, respectively. This pathway is little conserved, as observed in figure 4.

#### The Response of the DSB Subnetwork

This subnetwork is more tightly regulated than any other network, so the evolutionary conservation will be explained sequentially. The first step marks the damaged DSB sites, whereby the HR MRN-complex (*MRE11A-RAD50-NBN*) is recruited to the breaks, which in turns recruits the checkpoint *ATM* that becomes acetylated by *KAT5* (Sun et al. 2005). This process triggers *ATM* autophosphorylation (Lee et al. 2010) and the phosphorylation of *H2AFX* (Rogakou et al. 1998). Although the activator *KAT5* was identified in early eukaryotes, as well as *H2AFX*, the three components of the MRN-complex appeared at different evolutionary times. Although *RAD50* was detected in prokaryotes, *MRE11A* appeared in eukaryotes, and *NBN* appeared even later before the emergence of plants.

In the next phase, the phosphorylation of *H2AFX* produces a higher affinity for *MDC1* an essential mediator that orchestrates the further recruitment of additional factors (E3-ubiquitin ligases *RFN8*, *RNF168*, *UBE2N*, *HERC2*, and *RAD18*), triggering a complex cascade of histone ubiquitination at the DSB-flanking region. An important element in the assembly of these proteins is the SUMO E3-ligase *PIAS4*, an enzyme that acts on *RNF168* (Galanty et al. 2009) to provoke the restructuring of chromatin (Panier and Durocher 2009). There is little evolutionary conservation to this part of the pathway. Although E3-ubiquitinases are mostly of a distant origin (*UBE2N* is detected in prokaryotes, and both *RNF8* and *RAD18* are identified in early eukaryotes), their mediator at appropriate sites, *MDC1*, appeared very recently with the Vertebrata. The emergence of this gene is coupled to the later incorporation of E3-ubiquitin-ligases like *HERC2* (from animals), and much later *RNF168* in Chordata. *PIAS4*, which regulates *RNF168*, also appeared later in Vertebrata.

These ubiquitinated histones are essential hot-spots for recruiting different complexes involved in chromatin remodeling, and to promote HR repair. One such interaction is that between *RAD18* and the HR component *RAD50,* while the other is that between *PAXIP1* and *TP53BP1* (also involved in end resection: Bunting et al. 2010), both inducing chromatin remodeling. *RAD51* was identified in prokaryotes (see above in the HR pathway), and while *RAD18* and *PAXIP*1 first appeared in early eukaryotes, *TP53BP1* was identified much later in animals.

Many components of this pathway assemble with others that promote HR repair. For instance, *USP11* is a deubiquitination enzyme that interacts with *BRCA2* (Schoenfeld et al. 2004) and with *RAD51/TP53BP1* to promote HR (Wiltshire et al. 2010). *USP11* appeared in early eukaryotes, as did *BRCA2* (discussed earlier in the HR pathway).

A very important module of the DSB response that promotes HR repair, is the BRCA1-complex, which contains the HR components *BRCC36* (a deubiquitination enzyme), *BRCC45*, *BRCA1,* and the *BARD1* E3 ligase (Sobhian et al. 2007; Feng et al. 2009), as well as the other components like *UIMC1* (that binds to ubiquitinylated histones), and the proteins *FAM175A* and *BABAM1*. As described earlier, the role of SUMO E3-ligases is important. Moreover, in this module *PIAS1* interacts with *UIMC1* and *BRCA1* (Morris et al. 2009). This entire module is scarcely conserved and for instance, while the HR components *BRCC36*, *BRCA1* and *BRC45* are present in early eukaryotes, as well as *PIAS1*, *BABAM1,* and *BARD1* appeared later, before the split of plants. Moreover, the linker to ubiquitinated histones *UIMC1*, and *FAM175A* appeared much later before vertebrates. 

All together, these data indicate that this subnetwork was assembled in a modular way during evolution, reusing available components to produce a cross-talk between different pathways.

### Mapping Absences in the DDR Network

**Fig. 5.**
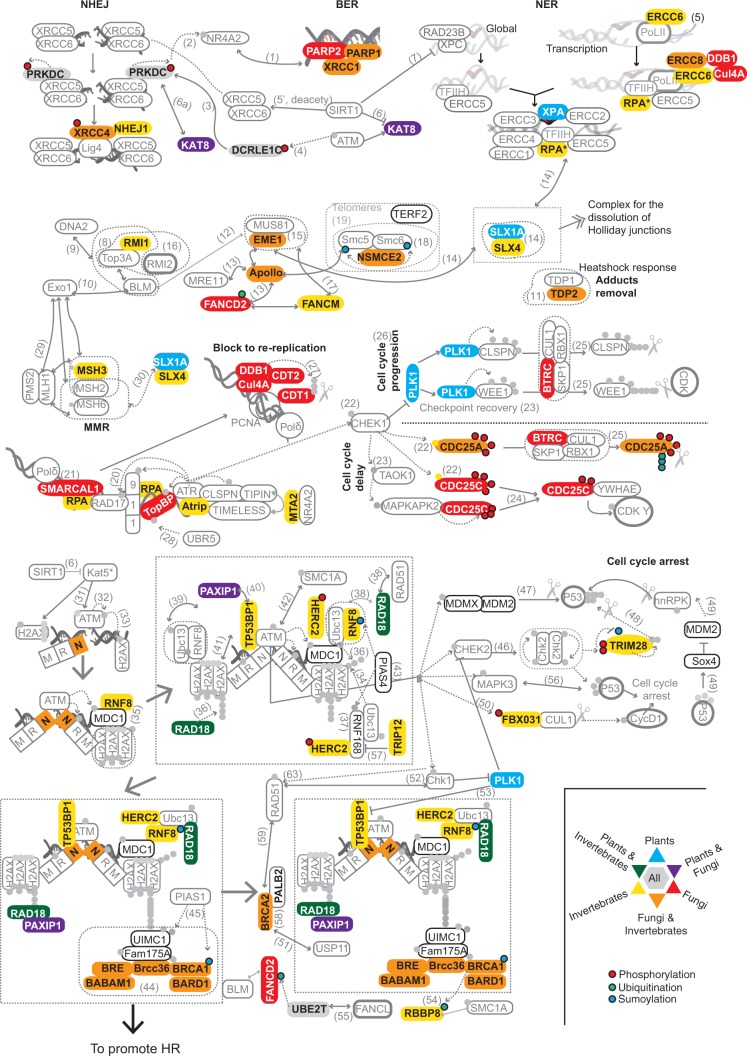
Mapping of gene absences in the DDR subnetworks. The figure illustrates the absence of DDR proteins in model organisms as mapped in figure 1. The absences were detected in plants, fungi, and invertebrates, and in combinations of these. For clarity, partial losses are not depicted (i.e., lost in *Saccharomyces cerevisiae* but present in *Sc. pombe*). For more detailed information regarding partial losses see figure 2. The numbers in brackets correspond to the PubMed identifiers as in fig. 1 (see supplementary text, Supplementary Material online).

Despite the overall conservation and the high level of conservation in distant eukaryotes, about 50 proteins were not found in model organisms. The lineages showing the largest number of absences were fungi and invertebrates (fig. 2). For example, 30 and 21 components were not found in *Saccharomyces cerevisiae* and *Schizosaccharomyces pombe,* respectively, and 29 and 26 components were absent from the nematode *Caenorhabditis elegans* and the arthropod *Drosophila melanogaster*, respectively (fig. 5).

#### Absences in the Global Repair Subnetwork

In the NHEJ pathway, *NHEJ1* was missing exclusively in *C. elegans* while it was conserved in the remaining species. *XRCC4* (that binds to DNA and *LIG4*) was missing in all fungi, nematodes, and arthropods, while it was present in basal animals. An important observation was that the BER pathway was entirely lost in model fungi (e.g., *S. cerevisiae*). Partial losses were observed for different lineages and for instance, *PARP1* had been lost in all the fungal species analyzed here, while *PARP2* was missing in all fungi except *Batrachochytrium dendrobatidis* (Bde, in draft state). *PARP2* was also absent in *C. elegans* and arthropods. *XRCC1* was also missing from all fungi and *C. elegans*.

The NER pathway accounted for many absences, although most of these were partial. For instance, *ERCC6* was lost in *D. melanogaster* and *RPA3* (a subunit of *RPA*) in *C. elegans*. Similarly, while *ERCC8* was lost in *D. melanogaster*, *C. elegans,* and *S. cerevisiae*, it was present in *Sc. pombe* and other worms. In addition, *XPA* was missing in plants. 

S*LX1A* and *SLX4* are important for the dissolution of joint DNA molecules and while the former is old, yet missing in plants, the latter was acquired in animals although it had been lost in nematodes and arthropods.

#### Absences in the RF

Among the components of the complexes at the RF, *SMARCAL1* was lost in model fungi and *MTA2* in *D. melanogaster*.

#### Absences in the Checkpoint Subnetwork

The entire block to rereplication pathway is lost in *S. cerevisiae*, while *CDT1* is the only missing component of this pathway in *Sc. pombe*. Regarding the checkpoints triggered at the forks, *ATRIP* is lost in *C. elegans* while *TOPBP1* in *S. cerevisiae.* In terms of cell cycle progression/delay, the checkpoint regulation component *BTRC* was lost in *S. cerevisiae* and *PLK1* is missing in plants. *S. cerevisiae* has *CDC25C*, which is missing in *Sc. pombe*. Conversely, *CDC25A* is lost in *S. cerevisiae* while it is present in *Sc. pombe*. Regarding cell cycle arrest pathways related to DDR, *TRIM28,* and *FBX031* were lost in model invertebrates while being represented in *Annelida* (*C. teleta*).

#### Absences in the DBS Subnetwork and the HR Pathway

As many components are related to both processes (fig. 1), we present them here together. At the break sites, the components of the BRCA1-complex *BABAM1*, *BRCC3*, *BRE*, *BARD1*, and even *BRCA1* were lost in model fungi (*S. cerevisiae* and *Sc. pombe*) and model invertebrates (*C. elegans* and *D. melanogaster*), although they were present in other species of the same lineages (fig. 2). From the MRN-complex, the *NBN* (“N” in figs. 1 and 5) was lost in *C. elegans* and model fungi. Other proteins recruited to such complexes, like *RNF8* and *TP53BP1*, were lost in model invertebrates while they were present in basal animals (*N. vectensis*), and *HERC2* was lost from *C. elegans*. *RAD18* is lost in *Arabidopsis thaliana* and model invertebrates, while its interacting *PAXIP1* is lost in *A. thaliana* and *S. cerevisiae*. *TRIP12* is lost in *C. elegans*.

### Phylogenetic Analyses of the DDR Proteins

Intrigued by these patterns of gain/loses, we conducted phylogenetic analyses of the genes having at least one component identified in early eukaryotes. We performed a total of 63 phylogenetic analyses, of which 48 were single gene trees and 15 were multigene trees containing 37 genes (supplementary table S1, Supplementary Material online). None of the multigene trees contained genes from the RF subnetwork. Of these 85 genes, 42 are from the GR subnetwork, 20 from the CHK subnetwork, 9 from the DSB subnetwork, and 7 were also from the RF subnetwork, as well as 7 for overlapping classes.

**Fig. 6.**
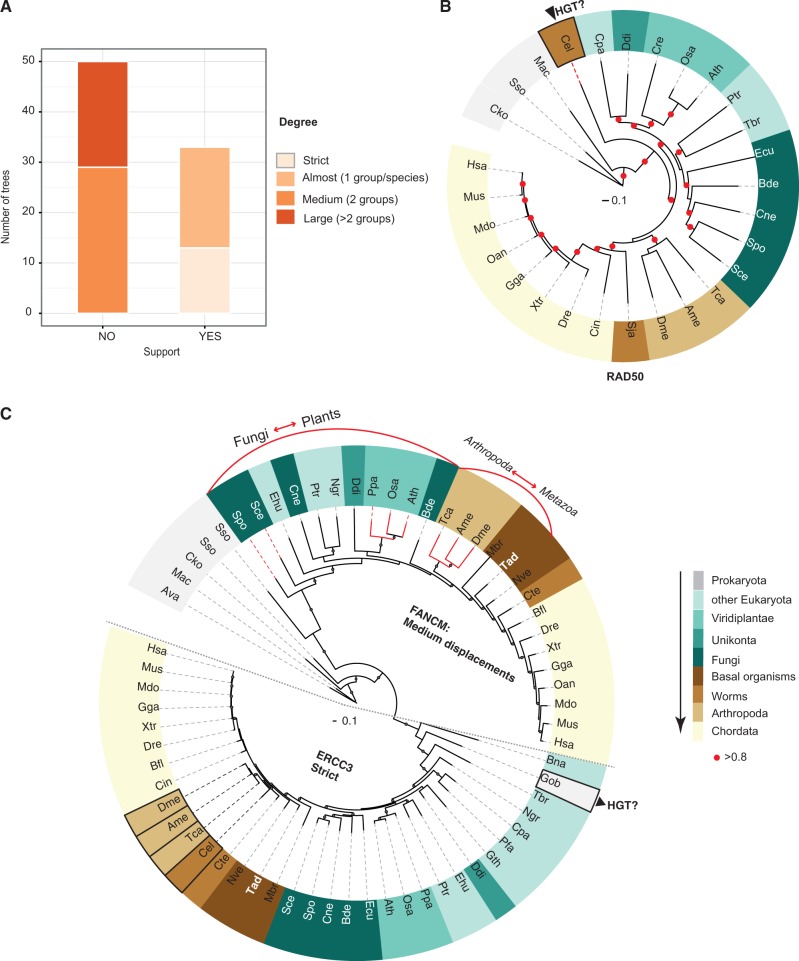
Summary of the phylogenetic analysis. (*A*) Summary of the findings. The plot indicates the agreement of DDR component gene trees with the species tree (depicted in supplementary fig. S1, Supplementary Material online). The degree of support is given by the number of groups involved in the misplacements that deviate from the species tree. For details about the individual trees, refer to supplementary table S1, Supplementary Material online. (*B*) Example of “Almost” indicates minor variations for *RAD50*, either a misplacement of either one group/species and/or observed artifacts. (*C*) Example of a “Strict” tree for *ERCC3* indicates that only minor variations are allowed (i.e., a potential HGT by a prokaryote). Example of “Medium” (*FANCM*) indicates misplacement of more than two species/groups.

Surprisingly, most of the trees did not support the species tree (fig. 6*A*) and only 13 strictly followed the species tree after still accommodating minor artifacts (like wrong sequences due to poor predictions, fragments, etc.). Only genes from the GR, CHK, and DSB subpathways were present in this class. There were 23 trees that followed the species trees with only minor variations, allowing for the misplacement of either one group/species and/or observed artifacts (i.e., *C. elegans* in *RAD50*; fig. 6*B*). The subnetworks in this class were GR, CHK, and DSB. The remaining trees displayed disagreements, whereby 29 trees had misplacements of up to 2 groups/species (medium) and 21 had large misplacements of species/groups. Although all the pathways were represented, two gene trees were considered to be unreliable (*RBX1* and *HUS1*).

Large simultaneous misplacements of more than one group involved plants and fungi in 15 cases out of 50, with the majority of the proteins showing this displacement from the GR subnetwork with all its pathways represented (supplementary fig. S2, Supplementary Material online). Within the 15 multigene trees, only in the *PIAS1/4* and *CDC25A*/C families did all their genes follow the species trees. Although certain members of the family followed the species tree (*ERCC3*), others did not (*FANCM*, fig. 6*C*). In four multigene trees, none of the genes from the same family followed the species tree (*MSH2/3/6*, *UBE2T/2N*, *ATM/R/PRKDC,* and *KAT5/KAT8*: supplementary fig. 2 for *MSH2/3/6*, *ATM/ATR/PRKDC*), whereas in the remaining trees at least one member of the family followed the species tree, either strictly or with only minor variations. 

The most variable organism was *C. elegans*, with 12 small (fig. 6*B*), 5 medium, and 16 large displacements. The basal animals *T. adhaerens* and *N. vectensis* were also frequently misplaced in 24 trees simultaneously.

In some cases, proteins from the same complex showed the same topology, which was not consistent with the species tree. For instance, *XRCC5* and *XRCC6* (supplementary fig. S3*A* and *B*, Supplementary Material online) are both components of the NHEJ pathway for which *C. elegans* groups with arthropods rather than with other worms. Another case is *RAD17* and *TOPBP1*, both present at the fork (supplementary fig. S3*C* and *D*, Supplementary Material online). The *PMS2* and *MLH1* gene family represented another example, producing proteins that dimerize to form MutLα in the MMR pathway and for which the sequences from *C. elegans* group close to distant eukaryotes (supplementary fig. S3*E*, Supplementary Material online).

In 5 of the 16 multigene trees, phylogenies helped to identify the incorrect orthology assignations, as was the case of *KAT8* and *KAT5* that belong to the same family. Most of the Eukaryota and plant *KAT8*, found as a bidirectional hit, are more likely to be *KAT5* proteins (fig. 2; supplementary fig. S2*D*, Supplementary Material online). Similarly, the *DCLRE1C/B* family also showed some inconsistencies produced by automatic orthology assignation, whereby *DCLRE1B* of *Physcomitrella patens*, Fungi and *Monosiga brevicollis* are rather *DCLRE1A* proteins (related homologs: supplementary fig. S2*E*, Supplementary Material online). Other examples include the *S. cerevisiae PIAS4* that is likely to be *PIAS1* and *Trypanosoma brucei PARP1* that might be *PARP2*. In 52 out of 65 trees, all the Chordata members followed the species tree. The exceptions were mainly due to *Ciona intestinalis* and *Branchiostoma floridae* grouping with more ancient groups, and misplaced incomplete sequences from *Ornithorhynchus anatinus* and *Monodelphis domestica.*

### Emergence of the Posttranslational Modifications of DDR Proteins

**Fig. 7.**
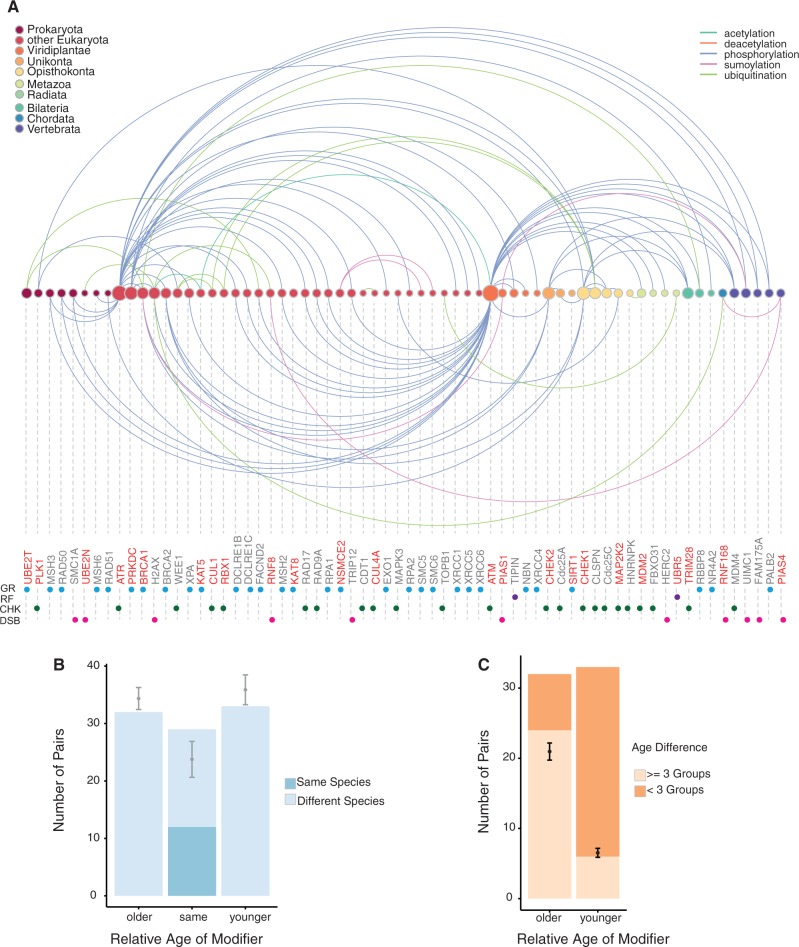
Analyses of posttranslational modifications in DDR proteins. (*A*) Arc-plot showing the interaction repertoire in function of the different ages (color circles). Colored edges over the circles indicate paired connections where the targets are younger than the modifiers or the connections are of the same age. Colored edges below the circles indicate pairs where the target is older than the modifier. Modification targets are named in gray, while the modifiers are in red. The diameter of the circles indicates the number of connections. Colored dots below the names indicate the subnetworks assigned to a particular component as in figure 1. The plot was generated with the R package (arc diagram, http://www.gastonsanchez.com/software.html, last accessed January 28, 2014). Dots below the names indicate subnetworks. (*B*) Distribution of interaction ages. Blue bars indicate number of modifier-target pairs in our data set according to the relative age of the modifier. Gray dots represent the average of the 1,000 randomized replicas and the bars the standard deviations. (*C*) Distribution of interaction pairs where the targets and modifiers are of different ages. Age difference was settled in three evolutionary jumps, where the age difference is more or less than 3 age groups. The black dots show 1,000 randomized replicas. There are fewer “small” (<3 groups) jumps than expected.

Finally, we set out to investigate the emergence of regulatory interactions among DDR components in a global manner, in particular those mediated by posttranscriptional modifications. Among the human DDR proteins in our data set, 53 were known targets of 24 modifiers, and targets and modifiers were represented in all the subnetworks. With the exception of the MMR pathway, all of them had at least one modifier, with the CHK subnetwork containing the largest number of modifiers (11 out of 24). Some modifiers produce self-modifications (*ATM*, *KAT8*, *PRKDC*, *TRIM28,* and *UBE2T*), as well as serving as targets of different modifiers. For instance, *CHEK2* is the target of *ATM* and a modifier of *BRCA1*. In total, there were 94 Target-Modifier pairs, 99 if we included self-modifications (fig. 7*A*). Regarding interaction types, the vast majority of posttranslational modifications involved phosphorylation (72 pairs), followed by ubiquitination (13 pairs), sumoylation (6 pairs), and acetylation (2 pairs: table 2). The most modified protein in the data set was *BRCA1*, which becomes phosphorylated, sumoylated and ubiquitinated. *H2AX* is also heavily and widely modified, although some specific residues that were ubiquitinated could not be identified precisely.

**Table 2.** Age Assignment of Interactions Associated with Posttranslational Modifications.

Target Gene	Target Network^a^	Modifier Gene	Modifier Network^a^	PTM^b^	Target Age^c^	Modifier Age^c^
*ATM*	CHK	*ATM*	CHK	P	V	V
*ATM*	CHK	*KAT5*	GNR|DSB	A	V	E
*BRCA1*	GR	*ATM*	CHK	P	E	V
*BRCA1*	GR	*ATR*	CHK	P	E	E
*BRCA1*	GR	*CHEK2*	CHK	P	E	U
*BRCA1*	GR	*PIAS1*	DSB	S	E	V
*BRCA1*	GR	*UBE2T*	DSB	U	E	Pr
*BRCA2*	GR	*CHEK1*	CHK	P	E	O
*BRCA2*	GR	*CHEK2*	CHK	P	E	U
*BRCA2*	GR	*ATM*	CHK	P	E	V
*BRCA2*	GR	*ATR*	CHK	P	E	E
*CDT1*	CHK	*CUL4*	GNR|CHK	U	E	E
*CHEK1*	CHK	*ATR*	CHK	P	O	E
*CHEK2*	CHK	*ATM*	CHK	P	E	V
*CLSPN*	CHK	*CHEK1*	CHK	P	O	O
*CLSPN*	CHK	*ATR*	CHK	P	O	E
*CLSPN*	CHK	*PLK1*	CHK	P	O	Pr
*CLSPN*	CHK	*CUL1*	CHK	U	O	E
*CLSPN*	CHK	*RBX1*	CHK	U	O	E
*DCLRE1B*	GR	*ATR*	CHK	P	E	E
*DCLRE1B*	GR	*ATM*	CHK	P	E	V
*DCR1C*	GR	*ATM*	CHK	P	E	V
*DCR1C*	GR	*PRKDC*	CHK	P	E	E
*EXO1*	GR	*ATR*	CHK	P	E	E
*FAM175A*	DSB	*ATM*	CHK	P	Vr	V
*FAM175A*	DSB	*ATR*	CHK	P	Vr	E
*FANCD2*	GR	*ATM*	CHK	P	E	V
*FANCL*	GR	*UBE2T*	GR	U	E	Pr
*FBX031*	CHK	*ATM*	CHK	P	M	V
*H2AFX*	DSB	*PRKDC*	CHK	P	E	E
*H2AFX*	DSB	*ATM*	CHK	P	E	V
*H2AFX*	DSB	*UBE2N*	CHK	U	E	Pr
*H2AFX*	DSB	*RNF168*	DSB	U	E	Ch
*H2AFX*	DSB	*RNF8*	DSB	U	E	E
*H2AFX*	DSB	*KAT5*	GNR|DSB	A	E	E
*HERC2*	DSB	*ATM*	DSB	P	M	V
*HNRPK*	CHK	*MDM2*	CHK	U	O	M
*MDM2*	CHK	*ATM*	CHK	P	M	V
*MDM4*	CHK	*CHEK1*	CHK	P	Vr	O
*MDM4*	CHK	*CHEK2*	CHK	P	Vr	U
*MDM4*	CHK	*ATM*	CHK	P	Vr	V
*MAPK3*	CHK	*MAPKAPK2*	CHK	P	E	O
*Cdc25A*	CHK	*CHEK1*	CHK	P	U	O
*Cdc25A*	CHK	*CHEK2*	CHK	P	U	U
*Cdc25C*	CHK	*CHEK1*	CHK	P	O	O
*Cdc25C*	CHK	*CHEK2*	CHK	P	O	U
*Cdc25C*	CHK	*MAPKAPK2*	CHK	P	O	O
*MSH2*	GR	*ATM*	CHK	P	E	V
*MSH2*	GR	*ATR*	CHK	P	E	E
*MSH3*	GR	*ATM*	CHK	P	Pr	V
*MSH3*	GR	*ATR*	CHK	P	Pr	E
*MSH6*	GR	*ATR*	CHK	P	Pr	E
*KAT8*	GR	*KAT8*	GR	A	E	E
*NBN*	GR|DSB	*ATM*	CHK	P	V	V
*NR4A2*	RFN|GR	*PRKDC*	CHK	P	B	E
*PALB2*	GR	*ATM*	CHK	P	Vr	V
*PALB2*	GR	*ATR*	CHK	P	Vr	E
*PRKDC*	GR	*PRKDC*	CHK	P	E	E
*RAD17*	RFN	*ATM*	CHK	P	E	V
*RAD17*	RFN	*ATR*	CHK	P	E	E
*RAD50*	GR|DSB	*ATM*	CHK	P	Pr	V
*RAD50*	GR|DSB	*ATR*	CHK	P	Pr	E
*RAD51*	GR|DSB	*CHEK1*	CHK	P	Pr	O
*RAD9A*	CHK	*ATM*	CHK	P	E	V
*RAD9A*	CHK	*ATR*	CHK	P	E	E
*RBBP8*	GR	*ATM*	CHK	P	B	V
*RBBP8*	GR	*BRCA1*	GR	U	B	E
*RPA1*	RFN	*ATM*	CHK	P	E	V
*RPA1*	RFN	*ATR*	CHK	P	E	E
*RPA2*	RFN	*PRKDC*	CHK	P	E	E
*RNF168*	DSB	*PIAS4*	DSB	S	Ch	Vr
*RNF8*	DSB	*PIAS4*	DSB	S	E	Vr
*SMC1A*	DSB	*ATM*	CHK	P	Pr	V
*SMC1A*	DSB	*ATR*	CHK	P	Pr	E
*SMC5*	GR	*NSMCE2*	GR	S	E	E
*SMC6*	GR	*NSMCE2*	GR	S	E	E
*TRIM28*	CHK	*ATM*	CHK	P	B	V
*TRIM28*	CHK	*CHEK2*	CHK	P	B	U
*TRIM28*	CHK	*CHEK1*	CHK	P	B	O
*TRIM28*	CHK	*TRIM28*	CHK	S	B	B
*TIPIN*	RFN	*ATR*	CHK	P	V	E
*TIPIN*	RFN	*ATM*	CHK	P	V	V
*TOPB1*	CHK	*UBR5*	RFN	U	E	M
*TRIP12*	DSB	*ATM*	CHK	P	E	V
*TRIP12*	DSB	*ATR*	CHK	P	E	E
*UBE2T*	GR	*UBE2T*	GR	U	Pr	Pr
*UIMC1*	DSB	*ATM*	CHK	P	Vr	V
*UIMC1*	DSB	*ATR*	CHK	P	Vr	E
*UIMC1*	DSB	*PIAS1*	DSB	S	Vr	V
*WEE1*	CHK	*PLK1*	DSB	P	E	Pr
*WEE1*	CHK	*CUL1*	DSB	U	E	E
*WEE1*	CHK	*RBX1*	CHK	U	E	E
*XPA*	GR	*ATM*	CHK	P	E	V
*XPA*	GR	*ATR*	CHK	P	E	E
*XPA*	GR	*SIRT1*	GR	deA	E	U
*XRCC1*	GR	*PRKDC*	GR	P	E	E
*XRCC4*	GR	*PRKDC*	GR	P	V	E
*XRCC5*	GR	*PRKDC*	GR	P	E	E
*XRCC6*	GR	*PRKDC*	GR	P	E	E

^a^DDR subnetwork types are GR, CHK, DSB, and RFN.

^b^Posttranslational modifications (PTMs): P is phosphorylation, U is ubiquitination, S is sumoylation, A is acetylation, and deA is deacetylation.

^c^Ages of targets and modifiers: B (Bilateria), Ch (Chordata), E (early Eukaryota), M (Metazoa) O (Opisthokonta), Pr (Prokaryota), U (Unikonta), Vr (Vertebrata), and V (Viridiplantae)*.*

To study the evolutionary origin of these interactions, we compared the relative ages of each member of the pair. In 29 cases, both target and modifier were in the same age group, whereas in 33 pairs the target is younger than the modifier and in 32 the opposite was true. Interestingly, in only 12 pairs where both the target and modifier of the same age, were homologs of the target and/or the modifier used to date the proteins when they were present in the same distant species. When we compared our observations with those obtained from 1,000 sets of randomized pairs, we observed that our DDR data set contains a larger number of pairs belonging to the same age group, and fewer pairs of different ages than expected (fig. 7*B*). We also found that interactions in which the modifier was older than the target tended to have greater differences in age than those in which the modifier belong to a more recent age group (fig. 7*C*). This fact could be attributed to most of the modifiers being of early origin, thereby limiting the distance to earlier targets. To check this possibility, we compared the distribution of interaction distances with that of a random set in which pairs were randomized, keeping the number of pairs in which the modifier was younger, older or the same age than the target constant. We found that in the observed set, the group of pairs in which the modifier is older than the target contained longer interactions than in the randomized set.

## Discussion

To date, a systematic functional and evolutionary analysis of the DDR network in humans has yet to be performed. In this study, we first assigned ages to genes and although no one optimal method has been established to define the age of a particular gene (Wolf et al. 2009; Capra et al. 2012), this concept proved to be reasonably accurate (Wolf et al. 2009). Indeed, discrepancies in the ages assigned to the genes probably reflect how different methods deal with gene gain and loss (Capra et al. 2012). These differences could also have been amplified due to the incompleteness of some genomes and by any potential lateral gene transfer.

Overall, our results indicate that while the origin of around 10% of the current human DDR components is traceable to the Prokaryota, the largest expansion of DDR components seems to have occurred during the appearance of the Eukaryota, where the network grew by ∼50–70%. We also show that the metabolic components of the network were dominant in the ancestral repertoire, with ∼70–80% of sensors and effectors present in distant eukaryotes, indicating that the ancestral network was enriched at both ends of any transduction pathway. Consistent with this, most of the oldest DDR components belong to the GR subnetwork, where effectors and sensors are the most represented functions. We also identified a strong conservation of complexes located at the RF, which also include a substantial fraction of Checkpoints components that were established early on in evolution, the oldest mostly being effectors. Additional regulatory layers came with the sequential incorporation of transducer and mediator functions that were particularly dominant in the DSB and RF subnetworks, and with the later emergence of regulators of existing pathways. For instance, *SIRT1* appeared in Unikonta and it is known to regulate both the NER (Ming et al. 2010) and the NHEJ (Sharma et al. 2010) pathways, which were already assembled at this age. Together, these results suggest the early emergence of the sensory and metabolic component of the DDR network, and the later appearance of the integrative regulatory element.

We did not identify the NHEJ pathway components in any of the prokaryotes analyzed, consistent with the lack of a canonical NHEJ pathway reported previously (Pitcher et al. 2007; Shuman and Glickman 2007). Indeed, alternative systems exist in pathogenic bacteria that are implicated in chromosomal translocation, such as A-EJ. Only structure-based methods can recover distant relationships between the *XRCC5/6* components and prokaryotic proteins, suggesting that these systems were in place early on in evolution (Chayot et al. 2010) and that maybe, much of the signaling sequence has been lost.

The poor conservation of the DSB subnetwork components that respond to *ATM* is remarkable. For instance, the central MRN complex (*MRE11A-RAD50-NBN1*) was assembled at different evolutionary steps and moreover, this network was built as an ensemble of distinct and available components (components involved in the HR repair pathway were incorporated to the foci). This overlap could be explained by the existence of an end-resection mechanisms that converts dsDNA into ssDNA enabling switching from error-prone to error-free repair (Cimprich and Cortez 2008), and where additional proteins from the DSB subnetwork participate, such as *TP53BP1* (Bunting et al. 2010). Components regulating important activities of the *P53* protein, such as the induction of cell-cycle arrest, senescence, or apoptosis, were recently incorporated in evolution, indicating that this subtle regulation of the cell cycle to overcome genome instability is very recent in evolution.

Although many checkpoint components generally appeared in early eukaryotes, those acting as linkers to trigger responses controlling cell cycle progression/delay appeared later on in evolution (like *CHEK1/2*). Thus, the assembly of the integrate control of the cell cycle toward progression or delay appeared before the split of animals and fungi, suggesting that plants use different routes. Conversely, the control of DNA rereplication is conserved, since in early eukaryotes it appears that this form of control maybe primordial, as supported by its presence in the NER pathway coupled to transcription.

Although true gene loss can only be assessed accurately in well-annotated and complete genomes, the frequency of gene loss in key parts of the subnetworks observed in complete genomes is generally quite remarkable, particularly in fungi and invertebrates. As these components were present in ancestral relatives like annelids (segmented worms), radiates such as *N. vectensis* (Cnidaria) and even Placozoa (*T. adhaerens*–Tad–the most primitive animal), we believe that these absences are generally due to gene loss. For model fungi, the entire BER pathway is lost in both species, while the control of DNA rereplication is only lost in its entirety in *S. cerevisiae*. Similarly, five components of the BRCA1-complex that promotes HR repair are also missing in model fungi and model invertebrates, while they are present in early eukaryotes. This indicates the possibility that this module has been lost independently at least twice: one in the line leading to fungi and other in that leading to invertebrates.

In cases of partial loss in a given pathway, it is possible that evolutionary unrelated genes will serve as functional analogs to perform the same function. This is consistent with results reported previously, showing that nematodes have lost several modules of regulatory networks (Srivastava et al. 2008) or fungi have incorporated novel lineage-specific proteins during their evolution (Wolf 2009, Fiedler 2009).

There were few gene duplication events in our data set, which alleviates the limitations of pairwise based methods to correctly detect orthologs using automatic approaches. Still, pairwise analysis may be affected when the sequence signal is lost, particularly in the presence of different protein domain arrangements, and/or the differential gene loss among orthologs that occurs in ancestral genomes. Specifically, it is difficult to identify horizontal gene transfer or to detect the most likely ortholog. Nevertheless, phylogeny helps in both cases as reported for eukaryotic-like transducers (Arcas et al. 2013) and for the *KAT8/5* family, respectively.

The major discrepancies between gene trees and species trees are probably due to difficulties to establish the true Tree of Life, and the existence of different evolutionary paces for different genes. Both these issues have a strong impact in alignment uncertainty, which has been reported to produce different phylogenetic trees (Wong et al. 2008). In this context, the most divergent genes are usually the most difficult to analyze. The ancestral origin of the vast majority of genes here could reflect extensive horizontal gene transfer, a process that has gained importance in eukaryotic organisms where it can be enhanced by particular lifestyles (Keeling and Palmer 2008).

In our data set, the recurrent inconsistency of *C. elegans* is particularly remarkable and it is unlikely to be an effect of long-branch attraction. Similar discrepancies have already been reported supporting alternative classifications for Nematoda and Arthropoda in the Ecdysozoa group (Aguinaldo et al. 1997) and for *T. adhaerens* (Srivastava et al. 2008) to lie closer to cnidarians. We have observed cases where genes from the same complexes may have been transferred in blocks (as in the case of some NHEJ components, supplementary fig. S3, Supplementary Material online), as trees show the same inconsistent topologies. Thus, our observations raise the question as to whether or not these well-established model organisms are useful to perform comparative studies of DDR outside the context of additional basal organisms.

The regulation of DDR by posttranslational modifications is still poorly understood (Polo and Jackson 2011). Our global approach suggests that a potential ancestral regulatory network was already in place before the Eukaryota split, to which additions were made at different steps. Like previous reports on protein–protein interactions (Capra et al. 2012), we found that posttranslational modifications appeared at the same ages more often than would be expected than if they were independent of age. Therefore, these gene pairs are likely to be coevolving.

In contrast to other studies, where proteins with regulatory activity are significantly younger than those showing catalytic activity (Capra et al. 2012), DDR transducers constitute ∼80% of the DDR functional tiers at Opisthokonta, suggesting that a functional network of posttranslational modifications had already been established. Although less than expected, we also observed large evolutionary jumps in the ages of each member of the pair, suggesting that the ability to modify and/or to be modified is quite flexible, reflecting remarkable plasticity in the regulatory network. Thus, a more promiscuous primordial repertoire would have exploded from a well-established interaction set, enabling the further acquisition of precise specificity in coevolving pairs.

To summarize, we have compiled the most complete network of human DDR pathways including regulatory aspects, and studied its emergence within a global evolutionary framework. The vast majority of these components have an ancient origin and while it is not surprising that the metabolic components of the network were predominant at early evolutionary times, so were the regulatory activities, even though they have subsequently expanded steadily during evolution. Repair based on the NHEJ pathway is probably the oldest part of the network, where similarities in prokaryotes can only be detected using sensitive structure-based methods, and where both canonical and noncanonical pathways are present. The newest acquisition is the response to DSB mediated by *ATM*, which seems to have grown by assembling existing components (i.e., the BRCA1-module) and including posttranslational modifications that affect protein complexes coupled to the regulation of the cell cycle. Entire pathways have been lost in some model organisms, and remarkable gene loss was observed in invertebrates. Moreover, gene loss in regulatory modules could have influenced the regulation of DDR in entire lineages (i.e., Nematoda vs. Annelida), where additional compensatory systems may have taken over.

## Materials and Methods

### Data Sets and Genome Sources

We examined the literature manually to compile a comprehensive list of DDR components from *H. sapiens*, retrieving 118 proteins (supplementary table S1, Supplementary Material online). To account for longer divergence times, we included three additional organisms in which DDR has been studied extensively: Ath (*A. thaliana*) with 122 proteins; Sce (*S. cervisiae*) with 91 proteins; and Eco (*Escherichia coli*) with 46 proteins (supplementary table S1, Supplementary Material online). To trace the DDR orthologs in the four organisms during evolution, we used the proteomes of 43 additional selected species available in the databases (supplementary table S2, Supplementary Material online). These include complete and incomplete proteomes, and they contain both predicted and confirmed peptide sequences. These data sets include 8 Eubacteria, 3 Archaea, and 36 Eukaryota. The organisms were grouped on the basis of previously defined phylogenetic studies (Roger and Simpson 2009) (see supplementary fig. S1, Supplementary Material online, for the phylogenetic trees of these species). When a particular proteome was available in different databases, the coverage was compared and the version containing the highest number of human DDR orthologs was chosen.

### Identification of Orthologs/Homologs

Each seed data set was used as a query list against 47 genomes to find orthologs using InParanoid (Remm et al. 2001) in its pairwise mode (supplementary table S3, Supplementary Material online). We first ran the program using the default parameters and then, slightly modifying the parameters to account for the large divergence times and to alleviate for the effects of using draft genomes. Accordingly, we made the confidence cutoff more stringent for in-paralog inclusion (from 0.05 to 0.25), we decreased the threshold for sequence overlap to obtain hits sharing common domains in distantly related organisms (from 0.5 to 0.4) and we slightly lowered the threshold of segment match coverage to obtain hits that share common domains (from 0.25 to 0.20). These modifications firstly aimed to avoid obtaining too many in-paralogs with very weak similarity to the main ortholog in distantly related organisms, and secondly, to avoid hits that share common domains in sequences that lie in unconserved regions, thereby always forcing the matched area to be longer than 40 % of the longer sequence. In all cases, the threshold e-value was –e 0.01. Different matrices were used in pairwise comparisons to account for different evolutionary distances: Blossum45 to compare prokaryotes, Blossum62 to compare eukaryotes, and Blossum80 to compare metazoans.

Given the few prokaryotic species included in these analyses, we extended the initial set to additional 41 prokaryotic species (supplementary table S2, Supplementary Material online). As the results in this extended set faithfully replicated the smaller set, we represent only the smaller set in the figures for clarity.

In the absence of clear homologs for *H. sapiens*, three additional seed organisms were used to extend the orthologous data sets when transitive matches were found (i.e., if a bidirectional hit of a human protein X [X_Hsa] was found in *S. cerevisiae* [X_Sce] but not in *Na. gruberi*, the existence of a bidirectional hit for X_Sce in *Na. gruberi* may point to a distant homolog of human [X_Hsa]). To confirm these relationships, protein domain architectures and length were checked. A whole list of proteins with orthologs in the 47 species is shown in supplementary table S3, Supplementary Material online.

### Classifications of DDR Network Genes Used in This Study

#### By Age Groups

We have defined 11 age groups in the represented species tree (which contains 47 species: supplementary fig. S1, Supplementary Material online), whereby: age group 1 includes homologs present in at least one representative of the main three suprakingdoms (across the 47 proteomes); age group 2 contains genes present in most Eukaryota (eight basal organisms, except organisms showing precise and particular lifestyles, like endosymbionts), but that are absent in Prokaryota; age group 3 includes proteins found in Viridiplantae (four organisms) but that are missing in older eukaryotes; age group 4 includes one Unikonta (Amoebozoa) representative; age group 5 points to the conservation in Opisthokonta (before the split of Fungi and Metazoa with five fungi and *M*. *brevicollis*); age group 6 is from the Metazoa (Placozoa representing *T. adhaerens* representing the most primitive animals); while age group 7 spans from Radiata and includes one cnidarian species (*N. vectensis*) to represent the different body plan symmetry; age group 8 is from the Bilateria (including flat worms and the Ecdysozoa group—Annelida and Arthropoda)*;* age group 9 includes the Chordata, age group 10 includes Vertebrata; and finally, age group 11 begins with Mammalia. We next classified each human gene to the age group of the most distant organism in which an ortholog for it was found based on our phylogenetic profile. For instance, as the more distant organisms where an *ATM* ortholog can be found are plants, we assigned *ATM* to the Viridiplantae age group to indicate that this protein was present in the common ancestor that included plants.

Alternatively, to determine whether or not the *H. sapiens* DDR network is enriched at certain ages, we used ProteinHistorian (Capra et al. 2012). We calculated enrichment using five different databases and two different methods (Wagner and Dollo parsimony) to account for the expected differences according to the different phylogenies and data sets. In all cases, *P* values were corrected with the Bonferroni test (table 1).

#### By Function

We used a broad classification widely used in the DDR field, as described previously (Petrini and Stracker 2003; Polo and Jackson 2011) and supported by the literature (supplementary table S1 [Supplementary Material online] and references therein): Effectors, Sensors, Transducers, and Mediators. These categories can be defined as follows: sensors typically detect alterations at the damage sites; mediators facilitate the interactions among components; transducers trigger signaling events (typically posttranslational modifications); and effectors perform physical actions (i.e., unwinding DNA, catalysis or attaching a molecule, etc.; Jackson and Bartek 2009). In this scheme, sensors and effectors represent the extremes of a given directed pathway, while alternative functions will be performed by the remaining classes, such as recruiting proteins (mediators) or triggering signals (transducers). In such settings, the same protein could fulfill more than one function (as described in the literature) and there are proteins involved in more than one repair pathway (supplementary table S1, Supplementary Material online).

### Phylogenetic Profiles

Phylogenetic profiles (Pellegrini et al. 1999) were constructed with the hits identified using pairwise InParanoid. These profiles were then clustered by hierarchical clustering with Cluster 3.0 (Eisen et al. 1998), using the Euclidean distance for the similarity metric and average linkage as the clustering method, which has proven successful elsewhere (Eisen and Hanawalt 1999). The proteins were sorted according to the species tree and blocks of stable proteins were obtained (fig. 2). The profiles were then used for gene content-based analyses, where each protein was considered as an independent hit to build the presence/absence matrix. Sequence similarities within the data set were not taken into account. To analyze the evolution of gene content in a given species, we used the Count package that contains different algorithms (Csuros 2010). In particular we used Wagner and Dollo parsimony to analyze the profiles of the sequences. The Dollo parsimony assumes a single appearance event per family (because gaining a gene is more rare than losing it), while Wagner parsimony allows multiple gain and loss events. As some of the genomes are in a draft stage (supplementary table S2, Supplementary Material online), we have not attempted to use probabilistic methods for ancestral reconstruction. To further represent the pace of growth according to the relative contribution of each age group on DDR components, we plotted the aggregated frequencies (normalized by group size) for each three methods: hierarchical clustering, Dollo parsimony, and Wagner parsimony (fig. 3). The figures were generated with R library ggplot2 (Wickham 2009).

### Mapping Evolution and Absences in the Human Network

Using the different pathways present in man (fig. 1), we mapped the age information extracted in previous sections to illustrate the evolutionary conservation of the components into the human network (fig. 4). In addition, reusing the same framework, we also mapped absences of DDR components in the lineages of model organisms (fig. 5).

### Phylogenetic Analyses

Homologous genes were aligned using the L-INS-I model in mafft (Katoh et al. 2005). The alignments were checked manually to identify potentially conflictive regions and only very large insertions occurring in a few sequences were excluded from any further analyses (probably prediction errors). In cases of minor domain variations (i.e., additional domains in particular lineages), only common domains were used to infer phylogenies (e.g., kinase domains in kinases). Once checked, the alignments were used as the input for probabilistic-based phylogeny studies (Ronquist and Huelsenbeck 2003) using MrBayes 3.1.2 with mpi parallel implementation. Only proteins with hits identified in prokaryotes or early eukaryotes (fig. 2) were analyzed. First, we classified trees as single-gene or multigene (evolutionary related). In a first approach, multigene families in-paralogs and paralogs extracted from Ensembl COMPARA (Flicek et al. 2013) were included in the phylogenetic analyses to ensure correct ortholog selection. For genes with minor domain variations (i.e., acquisition of a domain in the N-terminal or C-terminal regions within a particular lineage), only the common domains were used in the multiple alignments to infer their phylogenies. In a second approach, phylogenies were only run with the orthologous sequences. With some exceptions, we checked the COMPARA alignments available where the agreement was generally consistent. For instance, although COMPARA assigns the *PAXI1* and *MDC1* genes to the same family, there is no detectable sequence similarity between the two sequences except for a common BRCT protein domain. Given the promiscuity and the short length of the domain, we did not consider them as members of the same family. Jobs were run in our in-house cluster and in the Amazon’s Elastic Compute Cloud (EC2) using StarCluster utilities (http://star.mit.edu/cluster/index.html, last accessed January 28, 2014) for at least 5 million generations, and using mixed models of evolution. We discarded the initial 25% of the trees generated and further ensured that statistical convergence was reached. Consensus trees were generated from thousands of trees and clade probabilities were extracted directly from the samples. Gene trees were visualized with iTOL (Letunic and Bork 2007) and they were further compared with the species tree. To analyze the consensus trees, we manually estimated agreement with the species tree (fig. 6*A*). To assess the level of disagreement, we established the following criteria for misplacements, defining misplacement as the deviation from the expected topology (as depicted in supplementary fig. S1, Supplementary Material online) and in function of the number of groups involved in the misplacement (supplementary table S1, Supplementary Material online). Important groups here are Ecdysozoa (that includes the Nematoda, *C. elegans*, and Arthropoda: A), fungi (F) and plants (Pl), and basal metazoans (Basals, *T. adhaerens* and *M. brevicollis*). Small indicates either the presence of artifacts (wrongly predicted sequences, contaminations, etc.) and/or single species/group misplacement. Medium indicates misplacements involving two species/groups and/or artifacts, while Large indicates misplacements involving three or more species/groups and/or artifacts. We considered a tree to be well supported if the probability values for a cluster were more than 80% at deep branches.

### Analysis of Posttranslational Modifications

We extracted target-modifier pairs, and their sites, from the literature for *H. sapiens* (supplementary table S4, Supplementary Material online). Targets are proteins that are posttranslational modified in our DDR data set, while modifiers are the proteins in the same data set that perform the modification. The posttranslational modifications identified here involve phosphorylation, sumoylation, ubiquitination, and acetylation, as well as deubiquitination and deacetylation, as confirmed in some proteins by experimental data (supplementary table S4, Supplementary Material online). We next assigned ages to particular interactions, recording the ages of the individual proteins forming a pair (fig. 7*A* and table 2). To determine if our observations regarding the age of the interacting pairs shows any trends, we compared our observations with 1,000 random modification networks by shuffling the interacting pairs. For each random network, the relative age of the modifier with respect to the target was established and the frequency of the three possible outcomes was determined (younger, same age or older than the Modifier). Gray dots represent the average of the 1,000 replicas and the bars are the standard deviations. Self-modifications were excluded from these calculations (fig. 7*B* and *C*). To analyze if modifiers exert their action upon targets with a precise age, we compared our observations with randomized distributions reflecting different evolutionary jumps, where a jump reflects the interaction in which the members of the pair are of the same or different ages, regardless of the direction (fig. 7C). A plot indicating these interactions was generated with the R package (arc-diagram, http://www.gastonsanchez.com/software.html, last accessed January 28, 2014).

## Supplementary Material

Supplementary information, figures S1–S3, and tables S1–S4 are available at *Molecular Biology and Evolution* online (http://www.mbe.oxfordjournals.org/).

Supplementary Data

## References

[msu046-B1] Aguinaldo AM, Turbeville JM, Linford LS, Rivera MC, Garey JR, Raff RA, Lake JA (1997). Evidence for a clade of nematodes, arthropods and other moulting animals. Nature.

[msu046-B2] Ahn J, Urist M, Prives C (2004). The Chk2 protein kinase. DNA Repair (Amst)..

[msu046-B3] Allen C, Ashley AK, Hromas R, Nickoloff JA (2011). More forks on the road to replication stress recovery. J Mol Cell Biol..

[msu046-B4] Arcas A, Cases I, Rojas AM (2013). Serine/threonine kinases and E2-ubiquitin conjugating enzymes in Planctomycetes: unexpected findings. Antonie van Leeuwenhoek.

[msu046-B5] Arias EE, Walter JC (2007). Strength in numbers: preventing rereplication via multiple mechanisms in eukaryotic cells. Genes Dev..

[msu046-B6] Bae JB, Mukhopadhyay SS, Liu L, Zhang N, Tan J, Akhter S, Liu X, Shen X, Li L, Legerski RJ (2008). Snm1B/Apollo mediates replication fork collapse and S Phase checkpoint activation in response to DNA interstrand cross-links. Oncogene.

[msu046-B7] Bansbach CE, Betous R, Lovejoy CA, Glick GG, Cortez D (2009). The annealing helicase SMARCAL1 maintains genome integrity at stalled replication forks. Genes Dev..

[msu046-B8] Barnes DE, Lindahl T (2004). Repair and genetic consequences of endogenous DNA base damage in mammalian cells. Annu Rev Genet..

[msu046-B9] Bunting SF, Callen E, Wong N, Chen HT, Polato F, Gunn A, Bothmer A, Feldhahn N, Fernandez-Capetillo O, Cao L (2010). 53BP1 inhibits homologous recombination in Brca1-deficient cells by blocking resection of DNA breaks. Cell.

[msu046-B10] Capra JA, Williams AG, Pollard KS (2012). ProteinHistorian: tools for the comparative analysis of eukaryote protein origin. PLoS Comput Biol..

[msu046-B11] Chayot R, Montagne B, Mazel D, Ricchetti M (2010). An end-joining repair mechanism in *Escherichia coli*. Proc Natl Acad Sci U S A..

[msu046-B12] Ciccia A, Elledge SJ (2010). The DNA damage response: making it safe to play with knives. Mol Cell..

[msu046-B13] Cimprich KA, Cortez D (2008). ATR: an essential regulator of genome integrity. Nat Rev Mol Cell Biol..

[msu046-B14] Csuros M (2010). Count: evolutionary analysis of phylogenetic profiles with parsimony and likelihood. Bioinformatics.

[msu046-B17] Dunham I, Kundaje A, Aldred SF

[msu046-B15] Eisen JA, Hanawalt PC (1999). A phylogenomic study of DNA repair genes, proteins, and processes. Mutat Res..

[msu046-B16] Eisen MB, Spellman PT, Brown PO, Botstein D (1998). Cluster analysis and display of genome-wide expression patterns. Proc Natl Acad Sci U S A..

[msu046-B18] Fekairi S, Scaglione S, Chahwan C, Taylor ER, Tissier A, Coulon S, Dong MQ, Ruse C, Yates JR, Russell P (2009). Human SLX4 is a Holliday junction resolvase subunit that binds multiple DNA repair/recombination endonucleases. Cell.

[msu046-B19] Feng L, Huang J, Chen J (2009). MERIT40 facilitates BRCA1 localization and DNA damage repair. Genes Dev..

[msu046-B20] Fernandez-Capetillo O (2010). Intrauterine programming of ageing. EMBO Rep..

[msu046-B71] Fiedler D, Braberg H, Mehta M, Chechik G, Cagney G, Mukherjee P, Silva AC, Shales M, Collins SR, van Wageningen S (2009). Functional organization of the *S. cerevisiae* phosphorylation network. Cell.

[msu046-B21] Flicek P, Ahmed I, Amode MR, Barrell D, Beal K, Brent S, Carvalho-Silva D, Clapham P, Coates G, Fairley S (2013). Ensembl 2013. Nucleic Acids Res..

[msu046-B22] Fousteri M, Mullenders LH (2008). Transcription-coupled nucleotide excision repair in mammalian cells: molecular mechanisms and biological effects. Cell Res..

[msu046-B23] Galanty Y, Belotserkovskaya R, Coates J, Polo S, Miller KM, Jackson SP (2009). Mammalian SUMO E3-ligases PIAS1 and PIAS4 promote responses to DNA double-strand breaks. Nature.

[msu046-B24] Hentges P, Ahnesorg P, Pitcher RS, Bruce CK, Kysela B, Green AJ, Bianchi J, Wilson TE, Jackson SP, Doherty AJ (2006). Evolutionary and functional conservation of the DNA non-homologous end-joining protein, XLF/Cernunnos. J Biol Chem..

[msu046-B25] Jackson SP, Bartek J (2009). The DNA-damage response in human biology and disease. Nature.

[msu046-B26] Kadyrov FA, Dzantiev L, Constantin N, Modrich P (2006). Endonucleolytic function of MutLalpha in human mismatch repair. Cell.

[msu046-B27] Katoh K, Kuma K, Toh H, Miyata T (2005). MAFFT version 5: improvement in accuracy of multiple sequence alignment. Nucleic Acids Res..

[msu046-B28] Kazama Y, Ishii C, Schroeder AL, Shimada H, Wakabayashi M, Inoue H (2008). The *Neurospora crassa* UVS-3 epistasis group encodes homologues of the ATR/ATRIP checkpoint control system. DNA Repair (Amst)..

[msu046-B29] Keeling PJ, Palmer JD (2008). Horizontal gene transfer in eukaryotic evolution. Nat Rev Genet..

[msu046-B72] Kim H, Lee OH, Xin H, Chen LY, Qin J, Chae HK, Lin SY, Safari A, Liu D, Songyang Z (2009). TRF2 functions as a protein hub and regulates telomere maintenance by recognizing specific peptide motifs. Nat Struct Mol Biol..

[msu046-B30] Kimura S, Sakaguchi K (2006). DNA repair in plants. Chem Rev..

[msu046-B31] Kurosawa A, Adachi N (2010). Functions and regulation of Artemis: a goddess in the maintenance of genome integrity. J Radiat Res..

[msu046-B32] Lee JH, Goodarzi AA, Jeggo PA, Paull TT (2010). 53BP1 promotes ATM activity through direct interactions with the MRN complex. EMBO J..

[msu046-B33] Lees-Miller SP, Meek K (2003). Repair of DNA double strand breaks by non-homologous end joining. Biochimie.

[msu046-B34] Letunic I, Bork P (2007). Interactive Tree Of Life (iTOL): an online tool for phylogenetic tree display and annotation. Bioinformatics.

[msu046-B35] Li GM (2008). Mechanisms and functions of DNA mismatch repair. Cell Res..

[msu046-B73] Lukas J, Lukas C, Bartek J (2011). More than just a focus: The chromatin response to DNA damage and its role in genome integrity maintenance. Nat Cell Biol..

[msu046-B36] Marabotti A, Facchiano A (2009). When it comes to homology, bad habits die hard. Trends Biochem Sci..

[msu046-B37] Ming M, Shea CR, Guo X, Li X, Soltani K, Han W, He YY (2010). Regulation of global genome nucleotide excision repair by SIRT1 through *xeroderma pigmentosum C*. Proc Natl Acad Sci U S A..

[msu046-B38] Morris JR, Boutell C, Keppler M, Densham R, Weekes D, Alamshah A, Butler L, Galanty Y, Pangon L, Kiuchi T (2009). The SUMO modification pathway is involved in the BRCA1 response to genotoxic stress. Nature.

[msu046-B39] On T, Xiong X, Pu S, Turinsky A, Gong Y, Emili A, Zhang Z, Greenblatt J, Wodak SJ, Parkinson J (2010). The evolutionary landscape of the chromatin modification machinery reveals lineage specific gains, expansions, and losses. Proteins.

[msu046-B40] Panier S, Durocher D (2009). Regulatory ubiquitylation in response to DNA double-strand breaks. DNA Repair (Amst)..

[msu046-B41] Pellegrini M, Marcotte EM, Thompson MJ, Eisenberg D, Yeates TO (1999). Assigning protein functions by comparative genome analysis: protein phylogenetic profiles. Proc Natl Acad Sci U S A..

[msu046-B42] Peng L, Ling H, Yuan Z, Fang B, Bloom G, Fukasawa K, Koomen J, Chen J, Lane WS, Seto E (2012). SIRT1 negatively regulates the activities, functions, and protein levels of hMOF and TIP60. Mol Cell Biol..

[msu046-B43] Petrini JH, Stracker TH (2003). The cellular response to DNA double-strand breaks: defining the sensors and mediators. Trends Cell Biol..

[msu046-B44] Pitcher RS, Brissett NC, Doherty AJ (2007). Nonhomologous end-joining in bacteria: a microbial perspective. Annu Rev Microbiol..

[msu046-B45] Polo SE, Jackson SP (2011). Dynamics of DNA damage response proteins at DNA breaks: a focus on protein modifications. Genes Dev..

[msu046-B74] Potts PR, Yu H (2007). The SMC5/6 complex maintains telomere length in ALT cancer cells through SUMOylation of telomere-binding proteins. Nat Struct Mol Biol..

[msu046-B46] Raynard S, Bussen W, Sung P (2006). A double Holliday junction dissolvasome comprising BLM, topoisomerase IIIalpha, and BLAP75. J Biol Chem..

[msu046-B47] Reinhardt HC, Yaffe MB (2009). Kinases that control the cell cycle in response to DNA damage: Chk1, Chk2, and MK2. Curr Opin Cell Biol..

[msu046-B48] Remm M, Storm CE, Sonnhammer EL (2001). Automatic clustering of orthologs and in-paralogs from pairwise species comparisons. J Mol Biol..

[msu046-B49] Rogakou EP, Pilch DR, Orr AH, Ivanova VS, Bonner WM (1998). DNA double-stranded breaks induce histone H2AX P on serine 139. J Biol Chem..

[msu046-B50] Roger AJ, Simpson AG (2009). Evolution: revisiting the root of the eukaryote tree. Curr Biol..

[msu046-B51] Ronquist F, Huelsenbeck JP (2003). MrBayes 3: Bayesian phylogenetic inference under mixed models. Bioinformatics.

[msu046-B52] Santra MK, Wajapeyee N, Green MR (2009). F-box protein FBXO31 mediates cyclin D1 degradation to induce G1 arrest after DNA damage. Nature.

[msu046-B53] Schoenfeld AR, Apgar S, Dolios G, Wang R, Aaronson SA (2004). BRCA2 is ubiquitinated in vivo and interacts with USP11, a deubiquitinating enzyme that exhibits prosurvival function in the cellular response to DNA damage. Mol Cell Biol..

[msu046-B54] Sharma GG, So S, Gupta A, Kumar R, Cayrou C, Avvakumov N, Bhadra U, Pandita RK, Porteus MH, Chen DJ (2010). MOF and histone H4 acetylation at lysine 16 are critical for DNA damage response and double-strand break repair. Mol Cell Biol..

[msu046-B55] Shuman S, Glickman MS (2007). Bacterial DNA repair by non-homologous end joining. Nat Rev Microbiol..

[msu046-B56] Singh TR, Ali AM, Busygina V, Raynard S, Fan Q, Du CH, Andreassen PR, Sung P, Meetei AR (2008). BLAP18/RMI2, a novel OB-fold-containing protein, is an essential component of the Bloom helicase-double Holliday junction dissolvasome. Genes Dev..

[msu046-B57] Sobhian B, Shao G, Lilli DR, Culhane AC, Moreau LA, Xia B, Livingston DM, Greenberg RA (2007). RAP80 targets BRCA1 to specific ubiquitin structures at DNA damage sites. Science.

[msu046-B58] Srivastava M, Begovic E, Chapman J, Putnam NH, Hellsten U, Kawashima T, Kuo A, Mitros T, Salamov A, Carpenter ML (2008). The Trichoplax genome and the nature of placozoans. Nature.

[msu046-B59] Stracker TH, Usui T, Petrini JH (2009). Taking the time to make important decisions: the checkpoint effector kinases Chk1 and Chk2 and the DNA damage response. DNA Repair (Amst)..

[msu046-B60] Sun Y, Jiang X, Chen S, Fernandes N, Price BD (2005). A role for the Tip60 histone acetyltransferase in the acetylation and activation of ATM. Proc Natl Acad Sci U S A..

[msu046-B61] Theissen G (2002). Secret life of genes. Nature.

[msu046-B62] Toledo LI, Murga M, Zur R, Soria R, Rodriguez A, Martinez S, Oyarzabal J, Pastor J, Bischoff JR, Fernandez-Capetillo O (2011). A cell-based screen identifies ATR inhibitors with synthetic lethal properties for cancer-associated mutations. Nat Struct Mol Biol..

[msu046-B63] van Vugt MA, Gardino AK, Linding R, Ostheimer GJ, Reinhardt HC, Ong SE, Tan CS, Miao H, Keezer SM, Li J (2010). A mitotic P feedback network connects Cdk1, Plk1, 53BP1, and Chk2 to inactivate the G(2)/M DNA damage checkpoint. PLoS Biol..

[msu046-B64] Wakabayashi M, Ishii C, Inoue H, Tanaka S (2008). Genetic analysis of CHK1 and CHK2 homologues revealed a unique cross talk between ATM and ATR pathways in *Neurospora crassa*. DNA Repair (Amst)..

[msu046-B65] Wickham H (2009). ggplot2: elegant graphics for data analysis.

[msu046-B66] Williams RS, Williams JS, Tainer JA (2007). Mre11-Rad50-Nbs1 is a keystone complex connecting DNA repair machinery, double-strand break signaling, and the chromatin template. Biochem Cell Biol..

[msu046-B67] Wiltshire TD, Lovejoy CA, Wang T, Xia F, O'Connor MJ, Cortez D (2010). Sensitivity to poly(ADP-ribose) polymerase (PARP) inhibition identifies ubiquitin-specific peptidase 11 (USP11) as a regulator of DNA double-strand break repair. J Biol Chem..

[msu046-B68] Wolf YI, Novichkov PS, Karev GP, Koonin EV, Lipman DJ (2009). The universal distribution of evolutionary rates of genes and distinct characteristics of eukaryotic genes of different apparent ages. Proc Natl Acad Sci U S A..

[msu046-B69] Wong KM, Suchard MA, Huelsenbeck JP (2008). Alignment uncertainty and genomic analysis. Science.

[msu046-B70] Ye J, Lenain C, Bauwens S, Rizzo A, Saint-Léger A, Poulet A, Benarroch D, Magdinier F, Morere J, Amiard S (2010). TRF2 and apollo cooperate with topoisomerase 2alpha to protect human telomeres from replicative damage. Cell.

