# Supplementary material for: Emergence and Evolutionary Analysis of the Human DDR Network: Implications in Comparative Genomics and Downstream Analyses
Source: Mol Biol Evol. 2014 Jan 16;31(4):940–61. doi: 10.1093/molbev/msu046 (PMC3969565; doi:10.1093/molbev/msu046)
Supplement: Supplementary Data [file supp_31_4_940__index.html]

Emergence and evolutionary analysis of the human DDR network: implications in comparative genomics and downstream analyses — Emergence and Evolutionary Analysis of the Human DDR Network: Implications in Comparative Genomics and Downstream Analyses — Emergence and Evolutionary Analysis of the Human DDR Network: Implications in Comparative Genomics and Downstream Analyses — Supplementary Data 

# Emergence and Evolutionary Analysis of the Human DDR Network: Implications in Comparative Genomics and Downstream Analyses

## Supplementary Data

files

**Files in this Data Supplement:**

- Supplementary Data - pdf file
- Supplementary Data - txt file
- Supplementary Data - xlsx file
- Supplementary Data - xlsx file
- Supplementary Data - xlsx file
