# Supplementary material for: Emergence and Evolutionary Analysis of the Human DDR Network: Implications in Comparative Genomics and Downstream Analyses
Source: Mol Biol Evol. 2014 Jan 16;31(4):940–61. doi: 10.1093/molbev/msu046 (PMC3969565; doi:10.1093/molbev/msu046)
Supplement: Supplementary Data [file supp_msu046_Supp_Text_and_figures.pdf]

Supplementary Information

References for the illustrations depicted in figures 1, 4 and 5 of the main manuscript. The numbers in brackets in the figure indicate an association, the pubmed id and the reference.

(1)|12967653 (Caldecott 2003), (2)|21979916 (Malewicz et al. 2011), (3)|15456891 (Zhang et al. 2004), (4)|15468306 (Poinsignon et al. 2004), (5)|17334224 (Jeong et al. 2007), (6)|22586264 (Peng et al. 2012), (6a) (Sharma et al. 2010), (7)|21149730 (Ming et al. 2010), (8)|16595695 (Raynard et al. 2006), (9)|21252998 (Stracker and Petrini 2011), (10)|21325134 (Nimonkar et al. 2011), (11)|15141202 (Ostermeier et al. 2004), (12)|20655466 (Ye et al. 2010), (13)|18469862 (Bae et al. 2008), (14)|19596236 (Fekairi et al. 2009), (15)|12686547 (Ogrunc and Sancar 2003), (16)|18923083 (Singh et al. 2008), (17)|17289582 (Ciccia et al. 2007), (18)|17589526 (Potts and Yu 2007), (19)|19287395 (Kim et al. 2009), (20)|18594563 (Cimprich and Cortez 2008), (21)|19793861 (Bansbach et al. 2009), (22)|19230643 (Reinhardt and Yaffe 2009), (22)|19230643 (Reinhardt and Yaffe 2009), (23)|21278446 (Allen et al. 2011), (24)|21945648 (Gardino and Yaffe 2011), (25)|16600864 (Guardavaccaro and Pagano 2006), (26)|17084694 (Gewurz and Harper 2006), (27)|17344412 (Arias and Walter 2007), (28)|11714696 (Honda et al. 2002), (29)|18157157 (Li 2008), (30)|19596235 (Svendsen et al. 2009), (31)|16141325 (Sun et al. 2005), (32)|20010693 (Lee et al. 2010), (33)|9488723 (Rogakou et al. 1998), (34)|16427009 (Lou et al. 2006), (35)|19230794 (Panier and Durocher 2009), (36)|20023648 (Bekker-Jensen et al. 2010), (37)|19203579 (Doil et al. 2009), (38)|19396164 (Huang et al. 2009), (39)|15456759 (Jowsey et al. 2004), (40)|19124460 (Gong et al. 2009), (41)|22064073 (Yan et al. 2011), (42)|11877377 (Yazdi et al. 2002), (42)|19414588 (Wu et al. 2009), (43)|20016603 (Galanty et al. 2009), (44)|17525341 (Sobhian et al. 2007), (44)|19261746 (Shao et al. 2009), (44)|19261748 (Feng et al. 2009), (45)|20016594 (Morris et al. 2009), (46)|15279791 (Ahn et al. 2004), (47)|16163388 (Chen et al. 2005), (48)|19234109 (Pan et al. 2009), (49)|16360036 (Moumen et al. 2005), (50)|19412162 (Santra et al. 2009), (51)|15314155 (Schoenfeld et al. 2004), (52)|20233726 (Wiltshire et al. 2010), (53)|20126263 (van Vugt et al. 2010), (54)|16818604 (Yu et al. 2006), (55)|22615860 (Kelsall et al. 2012), (56)|22576881 (Heo et al. 2012), (57)|22884692 (Gudjonsson et al. 2012), (58)|16357213 (Kennedy and D'Andrea 2005), (59)|15800615 (Esashi et al. 2005), (60)|15665856 (Sorensen et al. 2005).

Supplementary Table 1. Summary of the results: table indicating the features assigned to each gene. Only complete information is provided for human sequences.

Supplementary Table 2. Summary of the 84 genomes used in this study. In the Organism ID column, NA indicates that although these prokaryotic genomes might have

been included in the screening, the findings can be represented by a smaller set and thus, they weren't included in the subsequent analyses.

Supplementary Table 3. Table indicating the orthologues found for *H. sapiens*, *A. thaliana*, *S. cerevisiae* and *E. coli*, using InParanoid and 4-way pairwise searches to examine the 47 proteomes.

Supplementary Table 4. Summary of the post-translational modifications collected for human proteins.

Supplementary figure legends

Supplementary Figure 1. Species tree. Only 11 prokaryotes out of 52 (Supplementary Table 2) are shown in the tree for clarity as these results are representative of the larger set. Left: Dashed black lines indicate the uncertainties when resolving the deep branches and the arrow at the bottom indicates the age in Millions of Years: green, bacteria; blue, *Archaea*; and black, *Eukaryota*. The red rectangles represent the age groups defined in this study, while the dashed boxes are the groups of genes defined elsewhere (Wolf et al. 2009): pink, endosymbionts (where only nucleomorph sequences are available); purple, parasites; orange, pathogens. # indicates complete sequence, while * indicates drafts. The species divergence times were extracted from http://www.treetime.org, using consensus estimates from the literature due to the fact that dates are not available for all the species.

Supplementary Figure 2. Phylogenetic trees of selected protein families. In all the trees the names in the boxes indicate the position of nematodes and arthropods, which always group outside the rest of the animals. **A. Family 35 (*ATR/ATM/PRKDC*)** illustrating how the phylogenies generated using only the common parts of the family (FAT-PI3_PI5_kin-FATC) serve to discriminate the overall domain architecture present in the other families. For instance, *A. thaliana* of *ATM* is the only representative containing a PWWP protein domain (indicated by *). The *ATR* and *ATM* of *C. elegans* do not contain additional domains, and they are grouped outside the expected placements, suggesting horizontal gene transfer. While *PKRDC* is missing in *C. elegans* it is present in other worms and basal organisms. **B. Family 02 (*MSH2/3/6*)**. This example illustrates the evolutionary models that differ from the species tree and between each other (dashed lines and triangles), as well as cases of domain shuffling, such as that of the PWWP protein domain present in the *MSH6* animals alone, except arthropods and nematodes (red arch), or in *O. sativa* (which could be an artifact due to it being a partial sequence). C. **Family 43 (*PARP1/2*).** This example illustrates protein domain shuffling in specific lineages, such as the SAP protein domain for *PARP2* in plants (indicated by *). **D.** Family 41. This example illustrates how automatic methods incorrectly identify orthologues. *KAT5* orthologues were identified as *KAT8* (boxed names). **E.** Family 40.

The inclusion of paralogues helps to identify real orthologues: *DCLRE1B* sequences for some species are in fact *DCLRE1A* (red lines).

Supplementary Figure 3. Phylogenetic analyses of proteins belonging to the same complex. The tree topologies are similar but distinct from the species tree. The boxed names indicate the situation in arthropods and nematodes: A and B are the *XRCC5/6* proteins from the NHEJ complex; D and C are *RAD17 and TOPB1* from the replication fork; and E is a multigene tree including the *PMS2* and *MLH1* proteins that dimerize in the mismatch repair pathway. Red arcs indicate misplacements.

Supplementary Figure 1

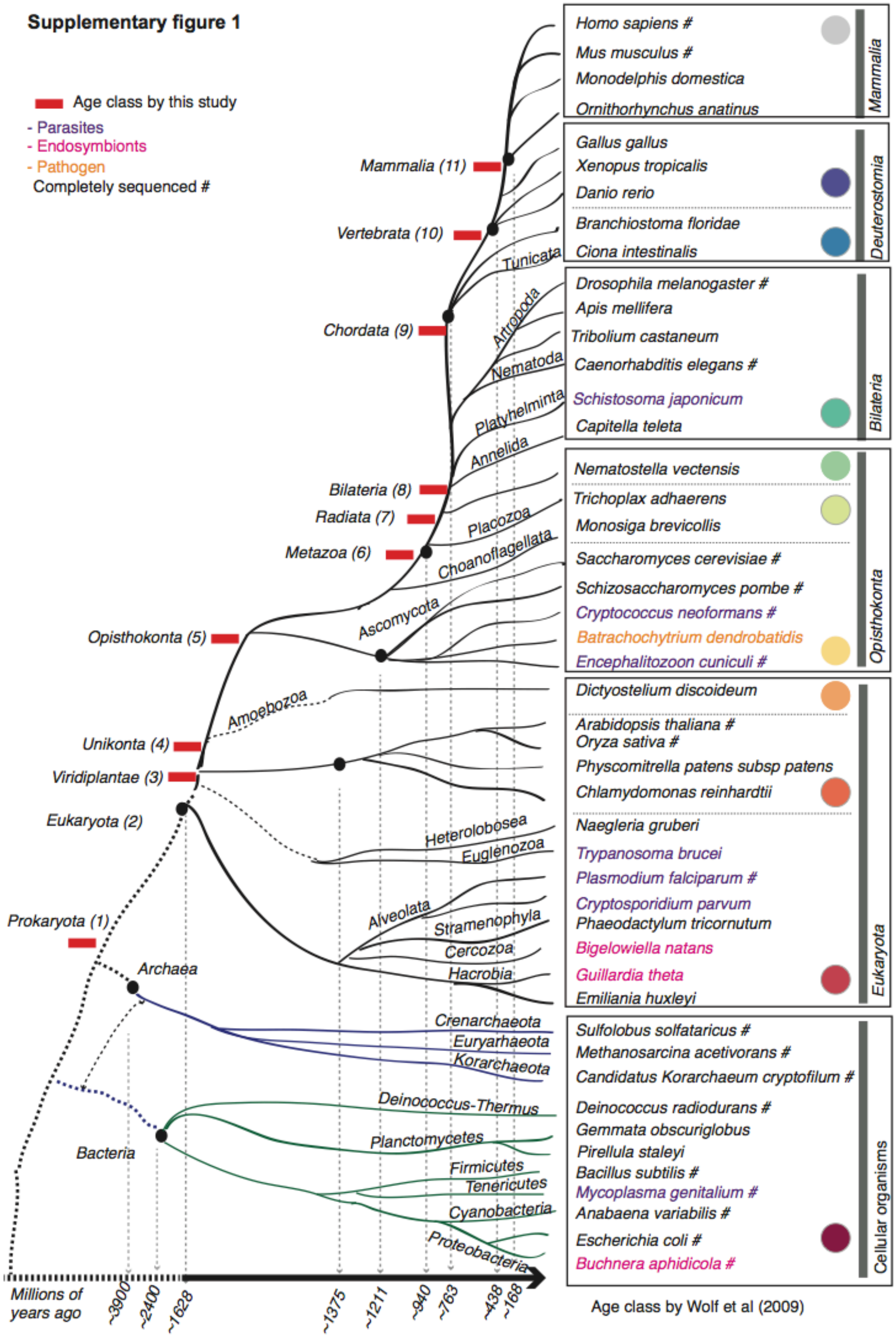

Supplementary Figure 2

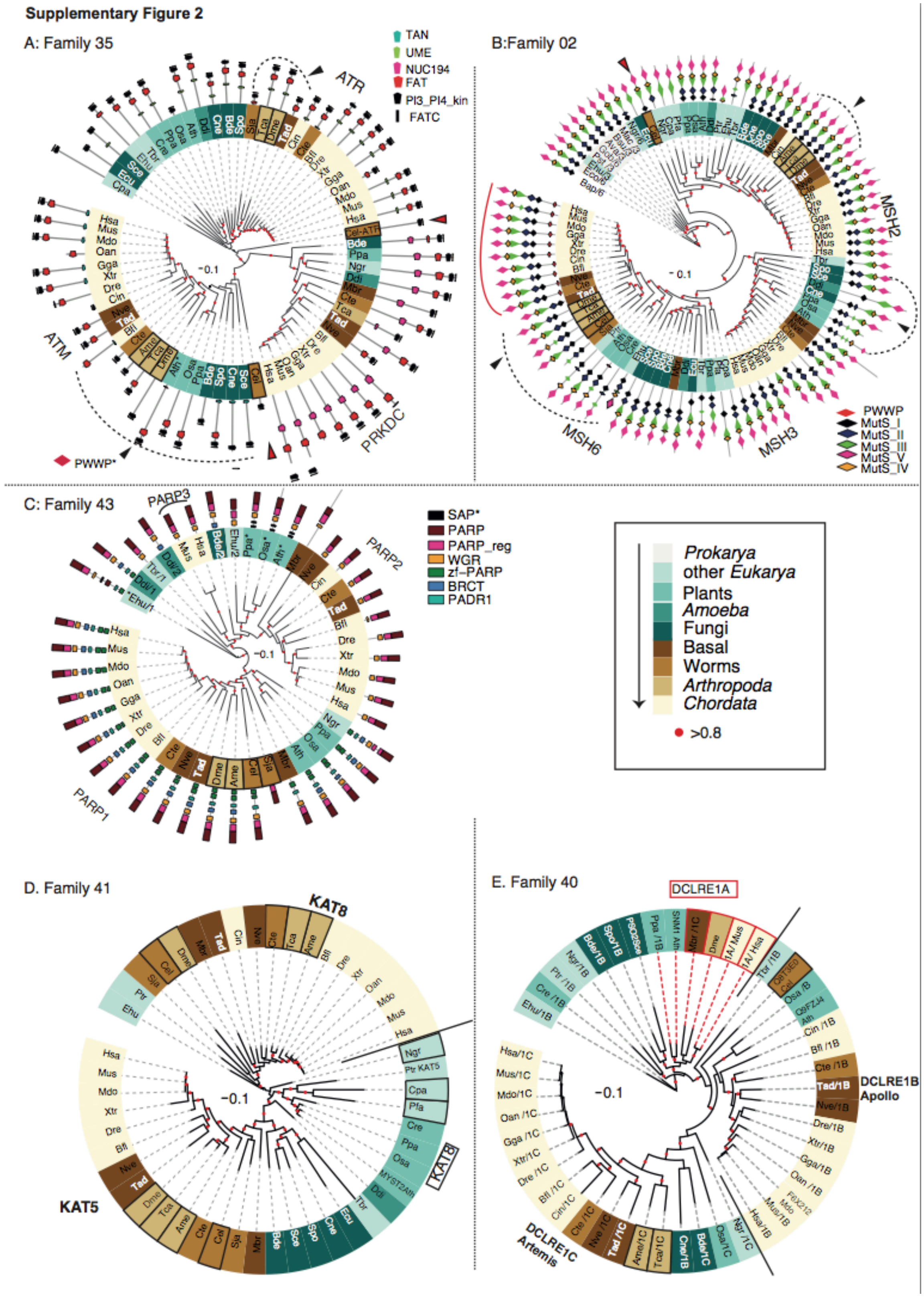

Supplementary Figure 3

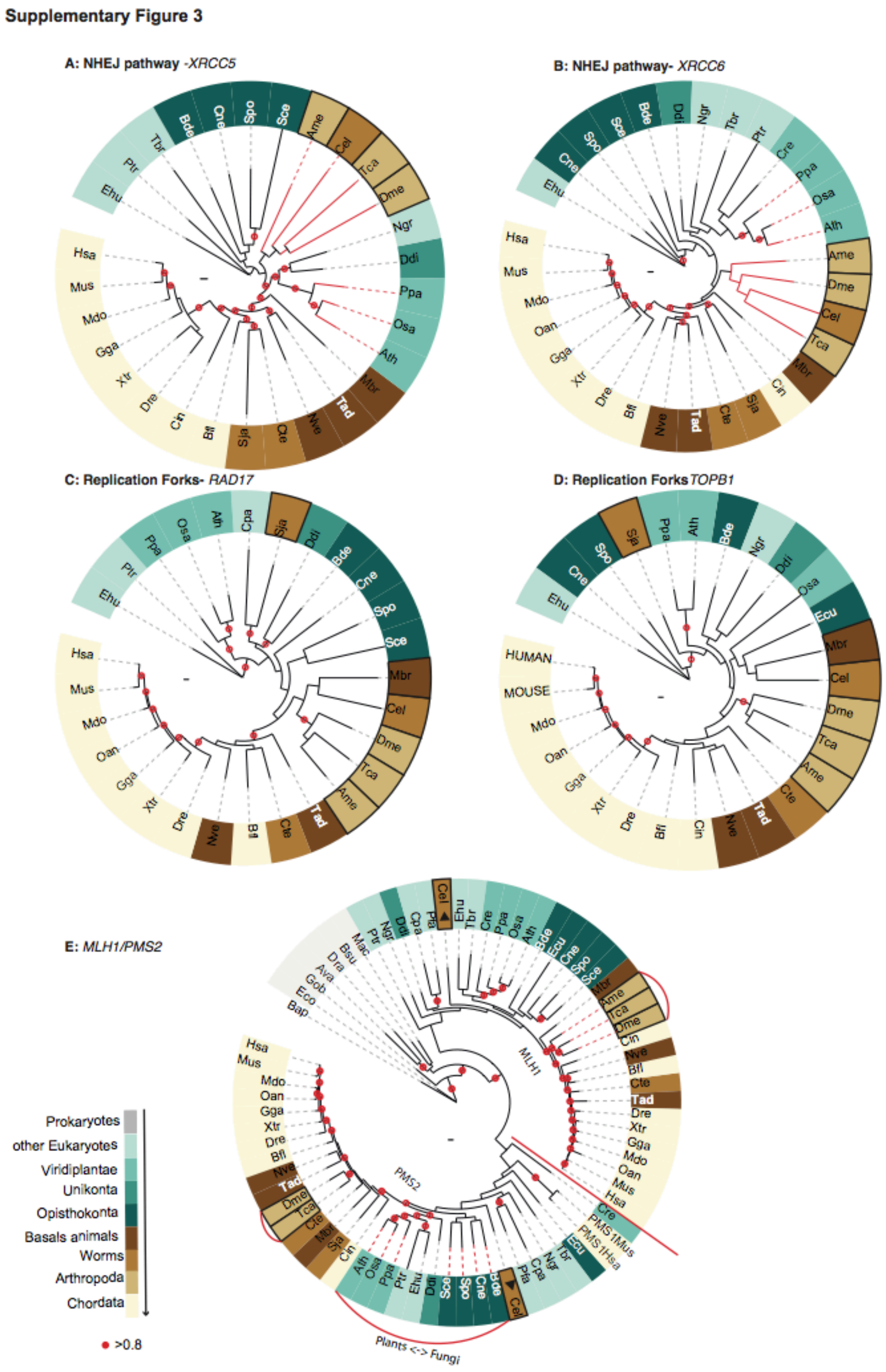

REFERENCES

Ahn J, Urist M, Prives C. 2004. The Chk2 protein kinase. *DNA Repair (Amst)* **3**(8-9): 1039-1047.

Allen C, Ashley AK, Hromas R, Nickoloff JA. 2011. More forks on the road to replication stress recovery. *J Mol Cell Biol* **3**(1): 4-12.

Arias EE, Walter JC. 2007. Strength in numbers: preventing rereplication via multiple mechanisms in eukaryotic cells. *Genes Dev* **21**(5): 497-518.

Bae JB, Mukhopadhyay SS, Liu L, Zhang N, Tan J, Akhter S, Liu X, Shen X, Li L, Legerski RJ. 2008. Snm1B/Apollo mediates replication fork collapse and S Phase checkpoint activation in response to DNA interstrand cross-links. *Oncogene* **27**(37): 5045-5056.

Bansbach CE, Betous R, Lovejoy CA, Glick GG, Cortez D. 2009. The annealing helicase SMARCAL1 maintains genome integrity at stalled replication forks. *Genes Dev* **23**(20): 2405-2414.

Bekker-Jensen S, Rendtlew Danielsen J, Fugger K, Gromova I, Nerstedt A, Lukas C, Bartek J, Lukas J, Mailand N. 2010. HERC2 coordinates ubiquitin-dependent assembly of DNA repair factors on damaged chromosomes. *Nat Cell Biol* **12**(1): 80-86; sup pp 81-12.

Caldecott KW. 2003. XRCC1 and DNA strand break repair. *DNA Repair (Amst)* **2**(9): 955-969.

Chen L, Gilkes DM, Pan Y, Lane WS, Chen J. 2005. ATM and Chk2-dependent phosphorylation of MDMX contribute to p53 activation after DNA damage. *EMBO J* **24**(19): 3411-3422.

Ciccia A, Ling C, Coulthard R, Yan Z, Xue Y, Meetei AR, Laghmani el H, Joenje H, McDonald N, de Winter JP et al. 2007. Identification of FAAP24, a Fanconi anemia core complex protein that interacts with FANCM. *Mol Cell* **25**(3): 331-343.

Cimprich KA, Cortez D. 2008. ATR: an essential regulator of genome integrity. *Nat Rev Mol Cell Biol* **9**(8): 616-627.

Doil C, Mailand N, Bekker-Jensen S, Menard P, Larsen DH, Pepperkok R, Ellenberg J, Panier S, Durocher D, Bartek J et al. 2009. RNF168 binds and amplifies ubiquitin conjugates on damaged chromosomes to allow accumulation of repair proteins. *Cell* **136**(3): 435-446.

Esashi F, Christ N, Gannon J, Liu Y, Hunt T, Jasin M, West SC. 2005. CDK-dependent phosphorylation of BRCA2 as a regulatory mechanism for recombinational repair. *Nature* **434**(7033): 598-604.

Fekairi S, Scaglione S, Chahwan C, Taylor ER, Tissier A, Coulon S, Dong MQ, Ruse C, Yates JR, 3rd, Russell P et al. 2009. Human SLX4 is a Holliday junction resolvase subunit that binds multiple DNA repair/recombination endonucleases. *Cell* **138**(1): 78-89.

Feng L, Huang J, Chen J. 2009. MERIT40 facilitates BRCA1 localization and DNA damage repair. *Genes Dev* **23**(6): 719-728.

Galanty Y, Belotserkovskaya R, Coates J, Polo S, Miller KM, Jackson SP. 2009. Mammalian SUMO E3-ligases PIAS1 and PIAS4 promote responses to DNA double-strand breaks. *Nature* **462**(7275): 935-939.

Gardino AK, Yaffe MB. 2011. 14-3-3 proteins as signaling integration points for cell cycle control and apoptosis. *Semin Cell Dev Biol* **22**(7): 688-695.

Gewurz BE, Harper JW. 2006. DNA-damage control: Claspin destruction turns off the checkpoint. *Curr Biol* **16**(21): R932-934.

Gong Z, Cho YW, Kim JE, Ge K, Chen J. 2009. Accumulation of Pax2 transactivation domain interaction protein (PTIP) at sites of DNA breaks via RNF8-dependent pathway is required for cell survival after DNA damage. *J Biol Chem* **284**(11): 7284-7293.

Guardavaccaro D, Pagano M. 2006. Stabilizers and destabilizers controlling cell cycle oscillators. *Mol Cell* **22**(1): 1-4.

Gudjonsson T, Altmeyer M, Savic V, Toledo L, Dinant C, Grofte M, Bartkova J, Poulsen M, Oka Y, Bekker-Jensen S et al. 2012. TRIP12 and UBR5 suppress spreading of chromatin ubiquitylation at damaged chromosomes. *Cell* **150**(4): 697-709.

Heo JI, Oh SJ, Kho YJ, Kim JH, Kang HJ, Park SH, Kim HS, Shin JY, Kim MJ, Kim M et al. 2012. ATM mediates interdependent activation of p53 and ERK through formation of a ternary complex with p-p53 and p-ERK in response to DNA damage. *Mol Biol Rep* **39**(8): 8007-8014.

Honda Y, Tojo M, Matsuzaki K, Anan T, Matsumoto M, Ando M, Saya H, Nakao M. 2002. Cooperation of HECT-domain ubiquitin ligase hHYD and DNA topoisomerase II-binding protein for DNA damage response. *J Biol Chem* **277**(5): 3599-3605.

Huang J, Huen MS, Kim H, Leung CC, Glover JN, Yu X, Chen J. 2009. RAD18 transmits DNA damage signalling to elicit homologous recombination repair. *Nat Cell Biol* **11**(5): 592-603.

Jeong J, Juhn K, Lee H, Kim SH, Min BH, Lee KM, Cho MH, Park GH, Lee KH. 2007. SIRT1 promotes DNA repair activity and deacetylation of Ku70. *Exp Mol Med* **39**(1): 8-13.

Jowsey PA, Doherty AJ, Rouse J. 2004. Human PTIP facilitates ATM-mediated activation of p53 and promotes cellular resistance to ionizing radiation. *J Biol Chem* **279**(53): 55562-55569.

Kelsall IR, Langenick J, MacKay C, Patel KJ, Alpi AF. 2012. The Fanconi anaemia components UBE2T and FANCM are functionally linked to nucleotide excision repair. *PLoS One* **7**(5): e36970.

Kennedy RD, D'Andrea AD. 2005. The Fanconi Anemia/BRCA pathway: new faces in the crowd. *Genes Dev* **19**(24): 2925-2940.

Kim H, Lee OH, Xin H, Chen LY, Qin J, Chae HK, Lin SY, Safari A, Liu D, Songyang Z. 2009. TRF2 functions as a protein hub and regulates telomere maintenance by recognizing specific peptide motifs. *Nat Struct Mol Biol* **16**(4): 372-379.

Lee JH, Goodarzi AA, Jeggo PA, Paull TT. 2010. 53BP1 promotes ATM activity through direct interactions with the MRN complex. *EMBO J* **29**(3): 574-585.

Li GM. 2008. Mechanisms and functions of DNA mismatch repair. *Cell Res* **18**(1): 85-98.

Lou Z, Minter-Dykhouse K, Franco S, Gostissa M, Rivera MA, Celeste A, Manis JP, van Deursen J, Nussenzweig A, Paull TT et al. 2006. MDC1 maintains genomic stability by participating in the amplification of ATM-dependent DNA damage signals. *Mol Cell* **21**(2): 187-200.

Malewicz M, Kadkhodaei B, Kee N, Volakakis N, Hellman U, Viktorsson K, Leung CY, Chen B, Lewensohn R, van Gent DC et al. 2011. Essential role for DNA-PK-mediated phosphorylation of NR4A nuclear orphan receptors in DNA double-strand break repair. *Genes Dev* **25**(19): 2031-2040.

Ming M, Shea CR, Guo X, Li X, Soltani K, Han W, He YY. 2010. Regulation of global genome nucleotide excision repair by SIRT1 through xeroderma pigmentosum C. *Proc Natl Acad Sci U S A* **107**(52): 22623-22628.

Morris JR, Boutell C, Keppler M, Densham R, Weekes D, Alamshah A, Butler L, Galanty Y, Pangon L, Kiuchi T et al. 2009. The SUMO modification pathway is involved in the BRCA1 response to genotoxic stress. *Nature* **462**(7275): 886-890.

Moumen A, Masterson P, O'Connor MJ, Jackson SP. 2005. hnRNP K: an HDM2 target and transcriptional coactivator of p53 in response to DNA damage. *Cell* **123**(6): 1065-1078.

Nimonkar AV, Genschel J, Kinoshita E, Polaczek P, Campbell JL, Wyman C, Modrich P, Kowalczykowski SC. 2011. BLM-DNA2-RPA-MRN and EXO1-BLM-RPA-MRN constitute two DNA end resection machineries for human DNA break repair. *Genes Dev* **25**(4): 350-362.

Ogrunc M, Sancar A. 2003. Identification and characterization of human MUS81-MMS4 structure-specific endonuclease. *J Biol Chem* **278**(24): 21715-21720.

Ostermeier GC, Miller D, Huntriss JD, Diamond MP, Krawetz SA. 2004. Reproductive biology: delivering spermatozoan RNA to the oocyte. *Nature* **429**(6988): 154.

Pan X, Zhao J, Zhang WN, Li HY, Mu R, Zhou T, Zhang HY, Gong WL, Yu M, Man JH et al. 2009. Induction of SOX4 by DNA damage is critical for p53 stabilization and function. *Proc Natl Acad Sci U S A* **106**(10): 3788-3793.

Panier S, Durocher D. 2009. Regulatory ubiquitylation in response to DNA double-strand breaks. *DNA Repair (Amst)* **8**(4): 436-443.

Peng L, Ling H, Yuan Z, Fang B, Bloom G, Fukasawa K, Koomen J, Chen J, Lane WS, Seto E. 2012. SIRT1 negatively regulates the activities, functions, and protein levels of hMOF and TIP60. *Mol Cell Biol* **32**(14): 2823-2836.

Poinsignon C, de Chasseval R, Soubeyrand S, Moshous D, Fischer A, Hache RJ, de Villartay JP. 2004. Phosphorylation of Artemis following irradiation-induced DNA damage. *Eur J Immunol* **34**(11): 3146-3155.

Potts PR, Yu H. 2007. The SMC5/6 complex maintains telomere length in ALT cancer cells through SUMOylation of telomere-binding proteins. *Nat Struct Mol Biol* **14**(7): 581-590.

Raynard S, Bussen W, Sung P. 2006. A double Holliday junction dissolvasome comprising BLM, topoisomerase IIIalpha, and BLAP75. *J Biol Chem* **281**(20): 13861-13864.

Reinhardt HC, Yaffe MB. 2009. Kinases that control the cell cycle in response to DNA damage: Chk1, Chk2, and MK2. *Curr Opin Cell Biol* **21**(2): 245-255.

Rogakou EP, Pilch DR, Orr AH, Ivanova VS, Bonner WM. 1998. DNA double-stranded breaks induce histone H2AX phosphorylation on serine 139. *J Biol Chem* **273**(10): 5858-5868.

Santra MK, Wajapeyee N, Green MR. 2009. F-box protein FBXO31 mediates cyclin D1 degradation to induce G1 arrest after DNA damage. *Nature* **459**(7247): 722-725.

Schoenfeld AR, Apgar S, Dolios G, Wang R, Aaronson SA. 2004. BRCA2 is ubiquitinated in vivo and interacts with USP11, a deubiquitinating enzyme that exhibits prosurvival function in the cellular response to DNA damage. *Mol Cell Biol* **24**(17): 7444-7455.

Shao G, Patterson-Fortin J, Messick TE, Feng D, Shanbhag N, Wang Y, Greenberg RA. 2009. MERIT40 controls BRCA1-Rap80 complex integrity and recruitment to DNA double-strand breaks. *Genes Dev* **23**(6): 740-754.

Sharma GG, So S, Gupta A, Kumar R, Cayrou C, Avvakumov N, Bhadra U, Pandita RK, Porteus MH, Chen DJ et al. 2010. MOF and histone H4 acetylation at lysine 16 are critical for DNA damage response and double-strand break repair. *Mol Cell Biol* **30**(14): 3582-3595.

Singh TR, Ali AM, Busygina V, Raynard S, Fan Q, Du CH, Andreassen PR, Sung P, Meetei AR. 2008. BLAP18/RMI2, a novel OB-fold-containing protein, is an essential component of the Bloom helicase-double Holliday junction dissolvasome. *Genes Dev* **22**(20): 2856-2868.

Sobhian B, Shao G, Lilli DR, Culhane AC, Moreau LA, Xia B, Livingston DM, Greenberg RA. 2007. RAP80 targets BRCA1 to specific ubiquitin structures at DNA damage sites. *Science* **316**(5828): 1198-1202.

Sorensen CS, Hansen LT, Dziegielewski J, Syljuasen RG, Lundin C, Bartek J, Helleday T. 2005. The cell-cycle checkpoint kinase Chk1 is required for mammalian homologous recombination repair. *Nat Cell Biol* **7**(2): 195-201.

Stracker TH, Petrini JH. 2011. The MRE11 complex: starting from the ends. *Nat Rev Mol Cell Biol* **12**(2): 90-103.

Sun Y, Jiang X, Chen S, Fernandes N, Price BD. 2005. A role for the Tip60 histone acetyltransferase in the acetylation and activation of ATM. *Proc Natl Acad Sci U S A* **102**(37): 13182-13187.

Svendsen JM, Smogorzewska A, Sowa ME, O'Connell BC, Gygi SP, Elledge SJ, Harper JW. 2009. Mammalian BTBD12/SLX4 assembles a Holliday junction resolvase and is required for DNA repair. *Cell* **138**(1): 63-77.

van Vugt MA, Gardino AK, Linding R, Ostheimer GJ, Reinhardt HC, Ong SE, Tan CS, Miao H, Keezer SM, Li J et al. 2010. A mitotic phosphorylation feedback network connects Cdk1, Plk1, 53BP1, and Chk2 to inactivate the G(2)/M DNA damage checkpoint. *PLoS Biol* **8**(1): e1000287.

Wiltshire TD, Lovejoy CA, Wang T, Xia F, O'Connor MJ, Cortez D. 2010. Sensitivity to poly(ADP-ribose) polymerase (PARP) inhibition identifies ubiquitin-specific peptidase 11 (USP11) as a regulator of DNA double-strand break repair. *J Biol Chem* **285**(19): 14565-14571.

Wolf YI, Novichkov PS, Karev GP, Koonin EV, Lipman DJ. 2009. The universal distribution of evolutionary rates of genes and distinct characteristics of

eukaryotic genes of different apparent ages. *Proc Natl Acad Sci U S A* **106**(18): 7273-7280.

Wu J, Prindle MJ, Dressler GR, Yu X. 2009. PTIP regulates 53BP1 and SMC1 at the DNA damage sites. *J Biol Chem* **284**(27): 18078-18084.

Yan W, Shao Z, Li F, Niu L, Shi Y, Teng M, Li X. 2011. Structural basis of gammaH2AX recognition by human PTIP BRCT5-BRCT6 domains in the DNA damage response pathway. *FEBS Lett* **585**(24): 3874-3879.

Yazdi PT, Wang Y, Zhao S, Patel N, Lee EY, Qin J. 2002. SMC1 is a downstream effector in the ATM/NBS1 branch of the human S-phase checkpoint. *Genes Dev* **16**(5): 571-582.

Ye J, Lenain C, Bauwens S, Rizzo A, Saint-Leger A, Poulet A, Benarroch D, Magdinier F, Morere J, Amiard S et al. 2010. TRF2 and apollo cooperate with topoisomerase 2alpha to protect human telomeres from replicative damage. *Cell* **142**(2): 230-242.

Yu X, Fu S, Lai M, Baer R, Chen J. 2006. BRCA1 ubiquitinates its phosphorylation-dependent binding partner CtIP. *Genes Dev* **20**(13): 1721-1726.

Zhang X, Succi J, Feng Z, Prithivirajsingh S, Story MD, Legerski RJ. 2004. Artemis is a phosphorylation target of ATM and ATR and is involved in the G2/M DNA damage checkpoint response. *Mol Cell Biol* **24**(20): 9207-9220.
